# Fruit and Vegetable Dietary Patterns and Mental Health in Women: A Systematic Review

**DOI:** 10.1093/nutrit/nuab007

**Published:** 2021-05-26

**Authors:** Dominika Guzek, Dominika Gła¸bska, Barbara Groele, Krystyna Gutkowska

**Affiliations:** Department of Food Market and Consumer Research, Institute of Human Nutrition Sciences, Warsaw University of Life Sciences, Warsaw, Poland; Department of Dietetics, Institute of Human Nutrition Sciences, Warsaw University of Life Sciences, Warsaw, Poland; Department of Dietetics, Institute of Human Nutrition Sciences, Warsaw University of Life Sciences, Warsaw, Poland; Department of Food Market and Consumer Research, Institute of Human Nutrition Sciences, Warsaw University of Life Sciences, Warsaw, Poland

**Keywords:** dietary patterns, fruits, juices, intake, Mediterranean diet, mental disorders, mental health, vegetables, vegetarian diet, women

## Abstract

**Context:**

Mental health may be influenced by some dietary patterns. Among common elements of beneficial patterns is high fruit and vegetable intake. However, no systematic review has been conducted to date, to our knowledge, that has assessed the influence of fruit and vegetable dietary patterns on a broad spectrum of mental health.

**Objective:**

We conducted a systematic review, using the PRISMA guidelines, of the observational studies analyzing the association between the dietary pattern of fruit and vegetables and the broad aspects of mental health in adult women.

**Data sources:**

The databases PubMed and Web of Science were searched, and additional manual search for observational peer-reviewed studies was conducted for studies published until June 2019.

**Data extraction:**

A total of 5911 studies were extracted and verified based on title and abstract for the inclusion criteria. All procedures were conducted independently by 2 researchers. The final number of included studies was 30. The review was structured around the type of observed outcome.

**Data analysis:**

The included studies had defined habitual intake associated with dietary patterns with the intake of specific fruit and/or vegetables, and/or fruit or vegetable products (eg, juices), as well as any aspect of the broad spectrum of general mental health. The Newcastle–Ottawa Scale was used to assess bias. The observed association was not stated in all the included studies; some of them revealed a reverse relationship, but only for the vegetarian/vegan diet. A vegetarian diet may be characterized by high consumption of fruits and vegetables, but it sometimes may not be properly balanced, due to excluded products. This may be the reason of observed situation.

**Conclusions:**

A general positive influence was observed for the dietary patterns characterized by high consumption of fruit and vegetables and of fruit or vegetable products by women.

**Systematic review registration:**

PROSPERO registration no. CRD42019138148.

## INTRODUCTION

Dietary patterns are defined as the specified quantity, proportion, and variety of food products consumed and the resultant quantity and proportion of nutrients in the habitual diet, including its multidimensional and dynamic characteristics.^1^ Because identifying the optimal dietary food-based recommendations for the prevention of chronic diseases is currently considered a public health priority, studies on dietary patterns are becoming vital.^2^ In recent years, recommendations and studies focusing on dietary patterns have been perceived as more practical than those focusing on nutrient intake, because the former allow promoting the consumption of healthy diets among individuals and population groups while providing specific information on food products that should be consumed.^3^

An increasing number of diseases are being proven to be prevented by adopting specific dietary patterns, and this has also been indicated in meta-analyses on the risk of cardiovascular diseases,^4^ chronic kidney disease,^5^ cancers (eg, colorectal,^6^ lung,^7^ endometrial,^8^ breast),^9^ frailty,^10^ neurodegenerative diseases,^11^ and attention deficit-hyperactivity disorder,^12^ among others. Moreover, specific dietary patterns have been attributed to a lower risk of cardiometabolic diseases^13^ and preterm birth,^14^ and a lower level of C-reactive protein.^15^

Among the conditions associated with mental health, depressive symptoms and depression were analyzed in a few studies and perinatal anxiety in 2 studies, but to our knowledge, none has been conducted thus far that assessed the influence of dietary patterns on a broad spectrum of mental health. In their systematic review and meta-analysis of studies conducted with community-dwelling adults, Lai et al^16^ showed that a diet high in fruit, vegetables, fish, and whole grains may be associated with a reduced risk of depression. Similarly, in another meta-analysis, Li et al^17^ indicated that a diet high in fruit, vegetables, whole grains, fish, olive oil, low-fat dairy products, and antioxidants, as well as low in animal foods, may be associated with a reduced risk of depression. In addition, in their systematic review and meta-analysis of observational studies, Nicolaou et al^18^ and Lassale et al^19^ compared the effects of some apparently healthy dietary patterns, based on Mediterranean Diet Score, the Healthy Eating Index, Alternative Healthy Eating Index, Dietary Inflammatory Index, and Dietary Approaches to Stop Hypertension diet, and stated that following those diets may lower the risk of development of depressive symptoms. Similar observations were reported by Quirk et al^20^ in their systematic review analyzing the effect of Mediterranean and traditional Norwegian diets in lowering the risk of depression; by Rahe et al^21^ in their study of the effect of the Mediterranean diet in lowering the risk of the onset of depression; and by Silva et al^22^ and Baskin et al^23^ in their systematic reviews on the effect of the dietary patterns interpreted as healthy in lowering the risk of perinatal anxiety and depression.

Analyzing the systematic reviews conducted so far on depression or depressive symptoms, it may be hypothesized that fruit and vegetables are among the main health-promoting components of the dietary patterns concluded to be beneficial and proven protective against depression. However, the only comprehensive study that analyzed a broad area of mental health in adults, conducted by Tarelho et al,^24^ was published just as an abstract of the 24th European Congress of Psychiatry, with no full paper provided, which indicates that more studies are necessary.

Taking this into account, as well as given the lack of studies on the general mental health of adults, we conducted a systematic review, using the Preferred Reporting Items for Systematic Reviews and Meta-Analyses (PRISMA) guidelines, of the observational studies analyzing the association between the dietary pattern of fruit and vegetables and the broad aspects of mental health in adult women. The systematic review verified whether the dietary pattern high in fruit and vegetables is associated with better mental health outcomes compared to other dietary patterns. The study was planned to be conducted specifically with women because the World Health Organization^25^ has indicated women are a vulnerable population due to a number of sex-related mental health disparities and has stated that there is a need for strategies dedicated especially to women.

## MATERIALS and METHODS

### Study design

The search for the studies was conducted based on the PRISMA guidelines.^26^ The systematic review was registered in the International Prospective Register of Systematic Reviews database (registration no. CRD42019138148) for assessment of fruit and vegetables intake (already published),^27^ as well as assessment of the fruit and vegetables dietary patterns.

The basic search strategy applied for the systematic review was the exclusion of the studies that (1) were not conducted in human adult populations, (2) assessed the intake of fruit and vegetables but did not include a defined dietary pattern, and (3) did not present the results observed for female participants separately from the male participants. The databases PubMed and Web of Science were searched for peer-reviewed observational studies published in English until June 2019, and an additional manual search was performed of the references of the studies that were found from these database searches.

### Inclusion/exclusion criteria

Studies were selected for inclusion and exclusion criteria, as presented for patient, intervention/exposure, comparator, outcome, and study design (PICOS) criteria (Table 1). The fruit and vegetable dietary patterns were indicated as a supposed reason and the broad aspects of mental health as a supposed consequence, so the included studies either specified such a causal nature or did not specify any reason and consequence, and just presented the coexisting intake and mental health; those studies that specified a reverse association (ie, mental health as a reason and dietary pattern as a consequence) were not included. All studies including information about dietary patterns including nonprocessed fruits or vegetables were included. The only required condition was the assessment of the intake of fruit, vegetables, and/or fruit or vegetable products (eg, juices) as a factor to differentiate populations and to define the dietary pattern. However, highly processed fruit and vegetable products (eg, ketchup, jam) were allowed as an element of the dietary pattern only if they were accompanied by nonprocessed fruit and/or vegetables. The assessment of intake had to have been associated with the documented habitual intake (with intervention studies excluded), but it was allowed to be conducted using any method, and the intake may have been specified either in grams or as a frequency of consumption (eg, number of servings), depending on the method applied by the authors of the study.

**Table 1:** PICOS criteria for inclusion and exclusion of studies

Parameter	Inclusion criteria	Exclusion criteria
Population	Adult women	Adult men, children, adolescents, patients diagnosed with any intellectual disabilities, patients diagnosed with any type of dementia, patients diagnosed with any eating disorders
Intervention/exposure	Participants characterized by defined habitual dietary patterns described as high in fruit and vegetable intake	No defined habitual dietary patterns described as high in fruit and vegetable intake
Comparison	Participants characterized by other defined habitual dietary patterns described as low to moderate in fruit and vegetable intake	Lack of compared group with defined habitual dietary patterns
Outcome	The aspects of mental health associated with any area of the broad spectrum of general mental health, among both healthy women and those with a physical disorder or disease	Patients assessed for cognitive function
Study design	Peer-reviewed articles published in English, including randomized controlled trials, randomized crossover trials, cohort studies, case-control studies, and cross-sectional studies	Articles not published in English; reviews, meta-analyses, expert opinions, letters to the editor, comments, studies in animal models, methodological articles, case reports, conference reports

The aspects of mental health that were allowed for the given studies were to be associated with any area of the broad spectrum of general mental health, among both healthy women and those with a physical disorder or disease. They had to have involved either subjective individual assessment (eg, participants’ own declarations) or assessment based on medical diagnosis. However, to provide reliable data, the following populations were excluded: (1) patients diagnosed with any intellectual disabilities, (2) patients diagnosed with any type of dementia assessed for cognitive function (as analyzed in the systematic review by Aridi et al^28^), and (3) patients diagnosed with any eating disorders. No additional criteria associated with country, location, ethnicity, or economic characteristics were required to be met by the studies for inclusion in the systematic review.

### Search strategy

**Figure 1:**
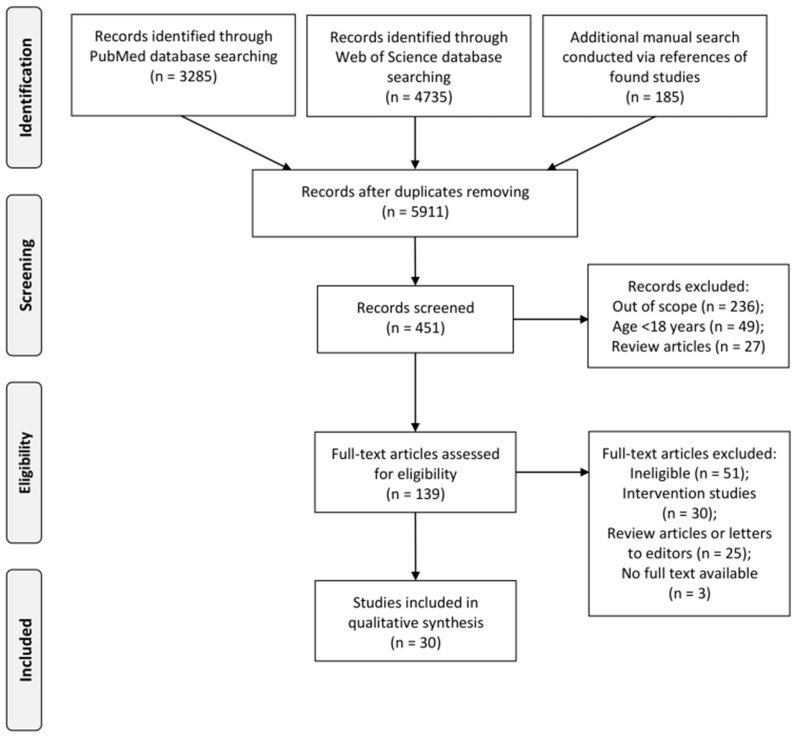
The search procedure applied for the systematic review and the flow of studies through each stage

The databases PubMed and Web of Science were searched for the relevant studies published until June 2019 (Table S1 in the Supporting Information online). An additional manual search of the references of the studies that were found in the databases was conducted to increase the possibility of including all the most important studies from the studied area. The search procedure applied for the systematic review and the flow of studies through each stage are presented in Figure 1.

The study extraction procedure was carried out independently by 2 researchers. The studies were screened independently by the researchers in 2 stages: (1) screening based on the title only and (2) screening based on the abstract (if the study was included on the basis of the title). If there was any disagreement between the researchers on any specific study, it was resolved by a discussion between themselves, or if needed, with a third researcher.

After the extraction procedure, the studies were qualified as potentially eligible. The full texts were retrieved from academic or other internet databases, or, if needed, from the corresponding authors through e-mail. To verify if the studies were potentially eligible to be included, an assessment of eligibility was carried out independently by 2 researchers. Similar to the previous stage, if there was any disagreement between the researchers on any specific study, it was resolved by a discussion between themselves, or if needed, with a third researcher.

### Data extraction

The data extraction procedure was carried out independently by 2 researchers. Similar to the previous stage, if there was any disagreement between the researchers on any specific study or data, it was resolved by a discussion between themselves or, if needed, with a third researcher. If any data were missing, the researchers contacted the corresponding authors to obtain them (in such situations, the data are presented in this systematic review as unpublished data provided by the authors of the study). The following data were extracted from the included studies: authors, study design, country/location, study group, time, number of participants, age, inclusion criteria, exclusion criteria, method of assessment of intake, characteristics of defined dietary patterns, outcome assessment, psychological measure, observations, and conclusions.

As indicated by the Cochrane recommendations for the tools for assessing the methodological quality or the risk of bias in nonrandomized studies,^29^ the Newcastle–Ottawa Scale (NOS)^30^ was chosen to be applied because it is commonly used.^31^ The studies were assessed for the following criteria: case–control studies: selection (scale: 0–4), comparability (scale: 0–2), and exposure (scale: 0–3); and cohort studies: selection (scale: 0–4), comparability (scale: 0–2), and outcome (scale: 0–3). After scoring, the results for each study were interpreted to be within the following categories: very high risk of bias (NOS points: 0–3), high risk of bias (NOS points: 4–6), and low risk of bias (NOS points: 7–9).^32^

The review was prepared on the basis of the data extracted and the assessment of the studies’ quality. The review was structured around the following types of outcomes: well-being, quality of life, positive and negative affect, self-esteem, anxiety, distress, depressive symptoms, depression, and suicide. Numerous various outcomes were included, with sometimes only a single study attributed to a defined outcome; therefore, it was not possible to summarize the results in the form of meta-analysis, which requires including comparable studies only (for 1 type of outcome). At the same time, if not only outcomes but also exposure (namely, dietary pattern), studied populations, and settings were not comparable, the included studies may not have been treated as sufficiently similar to reanalyze the data in the form of a meta-analysis. However, in the future, a meta-analysis would be valuable to include, but it should be conducted for various outcomes, not only for the depression/depressive symptoms, as it has been so far.^16–19^ As a result, the included studies were used to elaborate a synthesis of the findings structured around the type of outcome.

## RESULTS

The characteristics of studies of association between fruit or vegetable dietary pattern and mental health included to the systematic review are presented in Table 2.^33–62^ The characteristics of groups of women analyzed in the studies of association between fruit or vegetable dietary pattern and mental health included in this systematic review are presented in Table S2 in the Supporting Information online.^33–62^ The fruit or vegetable dietary patterns and mental health assessed in the studies included in this systematic review are presented in Tables 3 and 4,^33–62^ respectively. The detailed description of compared dietary patterns in the studies included in this systematic review is presented in Table S3 in the Supporting Information online.^33–62^

**Table 2:** The characteristics of studies of associations between fruit or vegetable dietary patterns and mental health included in this systematic review

Reference	Country/Location	Study design	Study group	Study period
Boldt et al (2018)^33^	Switzerland, Austria, Germany	Cross-sectional study within the Nutrition and Running High Mileage Study Step 2	Recreational runners	February–December 2015
Forestell and Nezlek (2018)^34^	United States	Cross-sectional study	Students in introductory psychology classes	Not specified
Gomes et al (2018)^35^	Brazil (Pelotas)	Cross-sectional, population-based study comprising a research consortium of Master’s degree students	Adults aged ≥ 60 years	2014
Li et al (2018)^36^	China (West Anhui)	Cross-sectional study within the Cohort of Elderly Health and Environment Controllable Factors	Adults aged ≥ 60 years	June–September 2016
Miyake et al (2018)^37^	Japan (Kyushu island, Okinawa prefecture)	Cross-sectional study within cohort of Kyushu Okinawa Maternal and Child Health Study	Pregnant women	April 2007 to March 2008
Teo et al (2018)^38^	Singapore	Cross-sectional study within Growing Up in Singapore Towards Healthy Outcomes Study	Postpartum women	Baseline: June 2009 to September 2010 (<13 weeks of pregnancy), followed up for 3 months postpartum
Adjibade et al (2018)^39^	France	Longitudinal, cross-sectional, population-based study within the Supplémentation en Vitamines et Minéraux Antioxydants study	Adults	1996–1997, 2007–2009
Baskin et al (2017)^40^	Australia	Cross-sectional study	Pregnant and early postpartum women	February 2010 to December 2011
Paskulin et al (2017)^41^	Southern Brazil	Cross-sectional study within Estudo do Consumo e do Comportamento Alimentar em Gestantes (Study of Food Intake and Eating Behaviors in Pregnancy)	Pregnant women	2006–2007
Sakai et al (2017)^42^	Japan	Cross-sectional population-based study within the Three-Generation Study of Women on Diets and Health	Women	April 2011, April 2012
Huddy et al (2016)^43^	Australia (Geelong, Melbourne)	Cross-sectional, population-based study within Melbourne Infant Feeding, Activity, and Nutrition Trial Extend Program	Mothers	2010–2011
Kim et al (2016)^44^	United States	Cross-sectional, population-based study within the National Health and Nutrition Examination Surveys	Adults aged 20–79 years	2007–2010
Liu et al (2016)^45^	Hong Kong	Cross-sectional study within the Soy Protein Study and the Whole Soy Study	Postmenopausal women aged 48–65 years	November 2007 to April 2008, December 2010 to January 2012
Hosseinzadeh et al (2016)^46^	Iran (Isfahan)	Cross-sectional population-based study within the Studying the Epidemiology of Psycho-Alimentary Health and Nutrition cohort	Adults aged 20–55 years	Not specified
Vilela et al (2015)^47^	Brazil (Rio de Janeiro)	Prospective cohort study	Women in midpregnancy to early postpartum	November 2009 to October 2011
Akbaraly et al (2013)^48^	United Kingdom	Prospective cohort study within the Whitehall II Study	Civil servants	1991–1993, 2003–2004, 2008–2009
Chocano-Bedoya et al (2013)^49^	United States	Prospective cohort study within the Nurses’ Health Study Study	Women	1980–2000
Ford et al (2013)^50^	United States	Cross-sectional study within Adventist Health Study-2 cohort	Adventist Church attendees	2002–2007
Nanri et al (2013)^51^	Japan	Prospective Study within The Japan Public Health Center–Based Study	Adults aged ≥40 years	1990–1993 and followed up until 2005
Rashidkhani et al (2013)^52^	Iran (Tabriz)	Cross-sectional study	Women	Not specified
Rienks et al (2013)^53^	Australia	Prospective cohort cross-sectional study within Australian Longitudinal Study on Women’s Health	Women	2001, 2004
Le Port et al (2012)^54^	France	Prospective cohort cross-sectional study within Gaz de France Electricite de France Cohort	Employees of France’s national Gas and Electricity Company	1989–2005
Chatzi et al (2011)^55^	Greece (Crete, Heraklion)	Prospective cohort study	Women	2007–2010
Jacka et al (2011)^56^	Norway	Cross-sectional, population-based study within the Hordaland Health Study	Adults aged 46–49 and 70–74 years	1997–1999
Okubo et al (2011)^57^	Japan (Osaka prefecture)	Prospective cohort study within Osaka Maternal and Child Health Study	Women	November 2001 to March 2003 and followed up for 2–9 months postpartum
Jacka et al (2010)^58^	Australia	Cross-sectional, population-based study within the Geelong Osteoporosis Study	Women	1994–1997, 2004–2008
Beydoun et al (2009)^59^	United States (Baltimore, Maryland)	Cross-sectional, population-based study within Healthy Aging in Neighborhoods of Diversity across the Life Span	Adults aged 30–64 years	Since November 4, 2004
Muñoz et al (2008)^60^	Spain (Gerona)	Cross-sectional, population-based study	Adults aged 35–74 years	2000–2005
Samieri et al (2008)^61^	France (Bordeaux)	Cross-sectional, population-based study within Three-City Study	Adults aged ≥ 65 years	2001–2002
Yannakoulia et al (2008)^62^	Greece (Attica region)	Cross-sectional, population-based study within the ATTICA Study	Adults	Not specified

**Table 3:** The fruit or vegetable dietary pattern in the studies included in this systematic review

Reference	Assessment	Fruit and vegetable dietary pattern
Boldt et al (2018)^33^	Question about the preferred diet	Vegetarian/vegan diet
Forestell and Nezlek (2018)^34^	General Eating Habits scale	Vegan; lacto-vegetarian; lacto-ovo-vegetarian; pesco-vegetarian; semi-vegetarian
Gomes et al (2018)^35^	EDQ-I	Healthy diet based on EDQ-I for higher consumption of healthy food groups
Li et al (2018)^36^	FFQ	Vegetable-based diet
Miyake et al (2018)^37^	Semiquantitative, comprehensive DHQ with 150 food items	Healthy dietary pattern
Teo et al (2018)^38^	3-Day dietary records	Soup, vegetables and fruits diet
Adjibade et al (2018)^39^	Repeated 24-h dietary records	Adherence to Mediterranean diet defined on the basis of relative Mediterranean diet score for high consumption of the desirable components
Baskin et al (2017)^40^	Cancer Council Victoria FFQ	Healthy dietary pattern
Paskulin et al (2017)^41^	FFQ with 88 food items	Varied dietary pattern
Sakai et al (2017)^42^	Comprehensive DHQ	Adherence to healthy diet defined on the basis of diet quality score, calculated on the basis of the intake of components recommended in the Japanese Food Guide Spinning Top, and sodium from seasonings
Huddy et al (2016)^43^	Cancer Council Victoria FFQ (Dietary Questionnaire for Epidemiological Studies, version 3.1)	Adherence to the 2013 Australian Dietary Guidelines assessed using the Dietary Guideline Index
Kim et al (2016)^44^	24-h dietary recall	Healthy dietary pattern
Liu et al (2016)^45^	FFQ with 85 food items	Whole-plant foods dietary pattern
Hosseinzadeh et al (2016)^46^	Dish-based semiquantitative FFQ with 106 food items	Lacto-vegetarian dietary pattern
Vilela et al (2015)^47^	FFQ with 82 food items	Healthy prepregnancy dietary pattern
Akbaraly et al (2013)^48^	FFQ with 127 food items	Adherence to healthy diet defined on the basis of the Alternative Healthy Eating Index score
Chocano-Bedoya et al (2013)^49^	FFQs with 61 and 131 food items	Prudent dietary pattern
Ford et al (2013)^50^	FFQ with >200 food items	Diet including Mediterranean foods
Nanri et al (2013)^51^	FFQ with 147 food items	Japanese dietary pattern
Rashidkhani et al (2013)^52^	FFQ with 125 food items	Healthy dietary pattern
Rienks et al (2013)^53^	FFQ with 101 food items	Cooked vegetables dietary pattern, fruit dietary pattern, Mediterranean style dietary pattern
Le Port et al (2012)^54^	FFQ with 35 food items	Healthy dietary pattern, traditional dietary pattern
Chatzi et al (2011)^55^	Rhea FFQ with 250 food items	Health conscious dietary pattern
Jacka et al (2011)^56^	FFQ with 169 food items	Healthy dietary pattern, traditional (Norwegian) dietary pattern
Okubo et al (2011)^57^	Comprehensive DHQ with 150 food items	Healthy dietary pattern
Jacka et al (2010)^58^	FFQ (Cancer Council Victoria dietary questionnaire) with 80 food items	(1) Traditional dietary pattern, Modern dietary pattern; (2) adherence to healthy diet defined on the basis of on diet quality score, calculated on the basis of the Australian national guidelines
Beydoun et al (2009)^59^	Two 24-h recalls	Adherence to healthy diet defined on the basis of 2005 USDA Healthy Eating Index
Muñoz et al (2008)^60^	FFQ with 165 food items	Adherence to Mediterranean diet defined based on Mediterranean diet score
Samieri et al (2008)^61^	FFQ	Healthy dietary pattern cluster
Yannakoulia et al (2008)^62^	EPIC-Greek semiquantitative FFQ with 156 food items	Healthful dietary pattern, vegetarian dietary pattern

Abbreviations: DHQ, Diet History Questionnaire; EDQ-I, Elderly Dietary Quality Index; FFQ, food frequency questionnaire.

**Table 4:** The mental health assessed in the studies included in this systematic review

Reference	Assessment	Psychological measure
Boldt et al (2018)^33^	Psychological well-being (ie, body image and appearance, negative feelings, positive feelings, self-esteem, spirituality/religion/personal beliefs, thinking, learning, memory and concentration)	World Health Organization Quality-of-Life Assessment- brief
Forestell and Nezlek (2018)^34^	(1) Depressive symptoms; (2) extraversion, agreeableness, conscientiousness, openness, and neuroticism	(1) CESD-20; (2) Big Five Inventory
Gomes et al (2018)^35^	(1) Depressive symptoms; (2) major depressive disorder	(1) Brazilian version of the GDS; (2) ICD-10, DSM-IV
Li et al (2018)^36^	Depression level	30-item Chinese revision of the GDS
Miyake et al (2018)^37^	Depressive symptoms	CESD-20
Teo et al (2018)^38^	(1) Depression; (2) anxiety	(1) Edinburgh Postnatal Depression Scale; (2) State-Trait Anxiety Inventory
Adjibade et al (2018)^39^	Depressive symptoms	CESD-20, French version
Baskin et al (2017)^40^	Depressive symptom	Edinburgh Postnatal Depression Scale
Paskulin et al (2017)^41^	Major depressive disorder, major depressive disorder in partial remission, dysthymia, panic disorder, generalized anxiety disorder, and bulimia nervosa	Patient Health Questionnaire from Primary Care Evaluation of Mental Disorders
Sakai et al (2017)^42^	Depressive symptoms	CESD-20, Japanese version
Huddy et al (2016)^43^	Depressive symptoms	CESD-10
Kim et al (2016)^44^	Symptoms of depression	Patient Health Questionnaire-9
Liu et al (2016)^45^	(1) Depressive symptoms; (2) nonspecific perceived stress; (3) self-esteem	(1) CES-D; (2) Perceived Stress Scale; (3) 10-item Rosenberg Self-Esteem Scale
Hosseinzadeh et al (2016)^46^	(1) Anxiety and depression; (2) psychological distress	(1) Hospital Anxiety and Depression Scale, Iranian version; (2) General Health Questionnaire
Vilela et al (2015)^47^	Anxiety symptoms	State-Trait Anxiety Inventory
Akbaraly et al (2013)^48^	Depressive symptoms	CESD-20
Chocano-Bedoya et al (2013)^49^	Psychological stress compared with well-being; depressive symptoms	5-item Mental Health Inventory scale
Ford et al (2013)^50^	Positive and negative affect	Positive and Negative Affect Schedule
Nanri et al (2013)^51^	Death from suicide	Death certificates with causes of death defined according to the ICD-10 (codes X60 to X84)
Rashidkhani et al (2013)^52^	Major depression	Structured Clinical Interview for DSM-IV Axis I Disorders
Rienks et al (2013)^53^	Depressive symptoms	CESD-10
Le Port et al (2012)^54^	Depressive symptoms	CESD-20
Chatzi et al (2011)^55^	Postpartum depression	Edinburg Postpartum Depression Scale
Jacka et al (2011)^56^	Depressive and anxiety symptoms	Hospital Anxiety and Depression Scale
Okubo et al (2011)^57^	Postpartum depression	Edinburg Postpartum Depression Scale, Japanese version
Jacka et al (2010)^58^	(1) Major depressive disorder, dysthymia, anxiety disorders; (2) Psychological symptoms	(1) Structured Clinical Interview for DSM-IV-TR Research Version, Non-Patient Edition; (2) 12-item General Health Questionnaire
Beydoun et al (2009)^59^	Depressive symptoms	CESD-20
Muñoz et al (2008)^60^	Health-related quality of life	The 12-Item Short Form Health Survey
Samieri et al (2008)^61^	Depressive symptoms	CES-D
Yannakoulia et al (2008)^62^	Levels of anxiety symptomatology	20-Item State-Trait Anxiety Inventory

Abbreviations: CESD-20, Center for Epidemiological Studies-Depression scale; DSM-IV, *Diagnostic and Statistical Manual of Mental Disorders, Fourth Revision*; GDS, Geriatric Depression Scale; ICD-10: *International Classification of Diseases, Tenth Revision.*

The observations and conclusions defined for the studies of association between fruit or vegetable dietary patterns and mental health included in this systematic review are presented in Table S4 in the Supporting Information online.^33–62^ The NOS scores for the categories of selection, comparability, and exposure/outcome for the assessment of the quality of included studies are presented in Table S5 in the Supporting Information online.^33–62^ The summary of observations and conclusions for the studies of association between fruit or vegetable dietary patterns and mental health included in this systematic review accompanied by the total NOS score are presented in Table 5.^33–62^

**Table 5:** Summary of observations and conclusions for the studies of association between fruit or vegetable dietary patterns and mental health included in this systematic review accompanied by the total Newcastle-Ottawa Scale score

Reference	Conclusions in terms of supporting fruit and vegetable dietary pattern recommendations instead of other patterns		Study quality^b^
	Association	Supporting/not supporting/inconclusive^a^	
Boldt et al (2018)^33^	Vegetarian or vegan diet: appropriate and equal alternative to an omnivorous diet	Inconclusive	3
Forestell and Nezlek (2018)^34^	Vegetarian or semi-vegetarian diet: higher level of openness to new experiences, higher risk of neurosis and depression	Not supporting	5
Gomes et al (2018)^35^	Low-quality diet: higher risk of depressive symptoms in elderly	Supporting	7
Li et al (2018)^36^	Vegetarian diets: higher risk of depressive symptoms	Not supporting	7
Miyake et al (2018)^37^	Healthy and Japanese dietary patterns: lower risk of depressive symptoms during pregnancy	Supporting	7
Teo et al (2018)^38^	Traditional-Indian-Confinement diet and soup-vegetables-fruits diet: lower risk of postpartum depression and postpartum anxiety symptoms	Supporting	7
Adjibade et al (2018)^39^	Mediterranean Diet: lower risk of incident depressive symptoms at midlife	Supporting	8
Baskin et al (2017)^40^	Poor diet quality: higher risk of depressive symptoms	Supporting	7
Paskulin et al (2017)^41^	Low consumption of fruits (common Brazilian dietary pattern): higher risk of mental disorders during pregnancy	Supporting	7
Sakai et al (2017)^42^	High diet-quality score: lower risk of depressive symptoms in young and middle-aged Japanese women	Supporting	7
Huddy et al (2016)^43^	Adherence to the Australian Dietary Guidelines: lower risk of mental health problems in first-time mothers	Supporting	7
Kim et al (2016)^44^	Healthy diet: lower risk of depression in women	Supporting	5
Liu et al (2016)^45^	Low intake of processed foods and/or a high intake of whole-plant foods: lower risk of depression and perceived stress	Supporting	6
Hosseinzadeh et al (2016)^46^	Increased intake of fruits, citrus fruits, vegetables, tomato: lower risk of psychological disorders	Supporting	7
Vilela et al (2015)^47^	Common Brazilian or healthy patterns: lower risk of anxiety symptoms from midpregnancy to early postpartum in Brazilian women	Supporting	6
Akbaraly et al (2013)^48^	Poor diet: higher risk of depression in women	Supporting	9
Chocano-Bedoya et al (2013)^49^	No clear association between dietary patterns and depression risk	Inconclusive	8
Ford et al (2013)^50^	Mediterranean diet: lower risk of negative affect in women	Supporting	5
Nanri et al (2013)^51^	Prudent dietary pattern: lower risk of suicide	Supporting	8
Rashidkhani et al (2013)^52^	Healthy dietary pattern: lower risk of depression in women	Supporting	6
Rienks et al (2013)^53^	Mediterranean-style dietary pattern: lower risk of depressive symptoms in mid-aged women	Supporting	9
Le Port et al (2012)^54^	Fruit/vegetable dietary patterns: lower risk of depressive symptoms	Supporting	8
Chatzi et al (2011)^55^	Healthy diet during pregnancy: lower risk for postpartum depression	Supporting	9
Jacka et al (2011)^56^	Better-quality diets: lower risk of depression	Supporting	6
Okubo et al (2011)^57^	No clear association between dietary patterns and postpartum depression risk	Inconclusive	7
Jacka et al (2010)^58^	High quality diet: lower risk of mental disorders	Supporting	7
Beydoun et al (2009)^59^	Unhealthy eating: higher risk of depression	Supporting	5
Muñoz et al (2008)^60^	Mediterranean diet: higher scoring for self-perceived health	Supporting	6
Samieri et al (2008)^61^	Fruit and vegetable dietary patterns: lower risk of depressive symptoms and better perceived health in older people	Supporting	8
Yannakoulia et al (2008)^62^	Fruit and vegetable dietary patterns: lower risk of anxiety	Supporting	6

a Supporting: fruit and vegetable dietary patterns were associated with lower risk of mental health problems; not supporting: fruit and vegetable dietary patterns were associated with higher risk of mental health problems; inconclusive: no clear association between fruit and vegetable dietary patterns and risk of mental health problems.

b Total score for the Newcastle-Ottawa Scale (NOS) is based on the following categories: very high risk of bias (0–3 NOS points), high risk of bias (4–6 NOS points), and low risk of bias (7–9 NOS points).^30^

## DISCUSSION

The conducted systematic review revealed a number of studies that assessed various dietary patterns, which were characterized by a high proportion of fruit and vegetables, as well as fruit and vegetable products. A majority of the included studies presented a pattern defined as a healthy or prudent diet, or as a diet based on specific dietary guidelines and recommendations, and characterized by a high intake of fruit and vegetables, either alone^61^ or in combination with cereals and whole grains,^35^^,^^40^^,^^42–44^^,^^50^^,^^56^^,^^58^^,^^59^^,^^62^ fish,^37^^,^^40^^,^^42^^,^^44^^,^^47^^,^^49^^,^^50^^,^^53^^,^^56^^,^^57^^,^^62^ nuts,^40^^,^^44^^,^^48^^,^^49^ or other products.^55^ In some studies, specific groups of fruit and vegetables were defined for this healthy pattern, such as dark green/green vegetables,^37^^,^^44^^,^^47^^,^^57^^,^^59^ yellow or orange vegetables,^37^^,^^44^^,^^57^^,^^59^ white vegetables,^57^ tomatoes,^44^ roots and tubers,^47^ soy,^48^ legumes,^37^^,^^47^^,^^49^^,^^53^^,^^59^ juices^47^^,^^53^^,^^59^ or salads,^5^ or they were were defined as both cooked and raw.^55^

Another important group of studies presented the Mediterranean dietary pattern, in which specific fruit and vegetables, such as garlic,^54^ peppers,^54^ salad greens,^54^ nonstarchy vegetables,^51^ and legumes^39^^,^^51^^,^^60^ were included, whereas potatoes^39^ and soy^51^ were excluded. The subsequent studies presented vegetarian/vegan dietary patterns,^33^^,^^34^^,^^46^^,^^62^ whereas some studies emphasized specific fruit and vegetables as included (eg, nonflatulent and flatulent vegetables, tomatoes, citrus fruits) within the pattern.^46^ Some studies also presented dietary patterns defined as traditional ones, for example, a traditional Japanese diet, including fruit, vegetables, seaweed, and soy products^52^; a traditional Norwegian diet, including fruit, vegetables, legumes, and numerous other products^56^; and just a traditional diet, being high in intake of fruit and fish,^55^ or fruit, vegetables, and some other products.^58^ One study presented a pattern called modern, which was high in intake of fruit and salads.^58^

The other group of included studies presented patterns focusing only on vegetables, fruit, or plant products. These patterns involved frequent consumption of vegetables,^36^ cooked vegetables (including cauliflower, cabbage, brussels sprouts, broccoli, and green beans),^54^ fruit (including strawberries, pineapple, melon, apricots, and mango),^54^ or fruit, vegetables, and whole grains.^45^ One study presented a dietary pattern described as varied, which included the intake of fruit, vegetables, tubers, and other products^41^; and another study presented a pattern called the “soup, vegetables and fruits” diet, which is high in the listed products.^38^

All the presented dietary patterns are characterized by a higher intake of fruit, vegetables, or fruit and vegetable products, compared with a typical diet. Following these patterns was linked with some common consequences for mental health in the vast majority of studies. Among the 30 studies included, 25 stated a positive influence of fruit and vegetable dietary pattern on mental health in women, independent of the assessed psychological outcome. These were the studies analyzing the influence on quality of life,^60^ positive and negative affect,^51^ self-esteem,^45^ anxiety,^38^^,^^46^^,^^47^^,^^56^^,^^58^^,^^62^ distress,^45^^,^^46^ depressive symptoms,^35^^,^^37^^,^^39^^,^^40^^,^^42–45^^,^^48^^,^^54–56^^,^^59^^,^^61^ depression,^35^^,^^38^^,^^41^^,^^46^^,^^49^^,^^53^^,^^58^ and suicide.^52^

Only 3 studies did not show such influence; in 2 of the 3, the results did not support a clear association between the dietary patterns and the risk of depression,^50^^,^^57^ and the third, conducted among endurance runners, revealed that the study group had a high quality of life regardless of the diet choice.^33^ Furthermore, 2 studies^34^^,^^36^ showed a reverse relationship, in which the dietary pattern including a high intake of fruit and vegetables was associated with worse mental health than the other patterns. It was observed that vegetarians and semi-vegetarians were more open to new experiences, but at the same time they exhibited more symptoms of neuroses and depression than did omnivores.^34^ In addition, it was found that in an elderly Chinese population, vegetarian diets may pose a greater risk of depressive symptoms than the other diets.^36^ It must be emphasized that the results mentioned for the indicated studies were formulated for specific population groups that follow not only a diet high in fruit and vegetables but also a vegetarian diet. This consequence of following a vegetarian diet was confirmed by the results of the study by Matta et al,^63^ which was conducted in a CONSTANCES cohort, among participants following various vegetarian diets. The study revealed that the development of depressive symptoms was associated with the exclusion of any food group from the diet, including animal products. Taking this into account, the indicated results^34^^,^^36^ should not be interpreted as a consequence of high fruit and vegetable intake but rather as a consequence of excluding some products from the diet.

The studies included in this systematic review were interpreted to be within the various categories of the risk of bias, based on the NOS score; therefore, it should be indicated that some studies were defined as having a low risk of bias (or even a very low risk of bias, indicated by a maximum NOS score) and their results should be considered as the most prominent. The risk of bias is associated with the likelihood that the design or conduct of the study will give rise to misleading results and, therefore, the studies with the lowest risk of bias should be the basis of the conclusions. It should be mentioned that in the present study, the results of all included references are quite similar. However, the observations from the studies with a higher risk of bias may be interfered and potentially may not provide accurate conclusions. Within the studies with the lowest risk of bias, it was observed that a high-quality, healthy, and properly balanced diet with a large amount of fruit and vegetables may be associated with a reduced risk of anxiety,^38^ mental disorders,^41^^,^^43^^,^^46^^,^^58^ and depressive symptoms in women,^35^^,^^37^^,^^39^^,^^40^^,^^42^^,^^48^^,^^49^^,^^54^^,^^55^^,^^61^ and of their consequences, such as suicide.^52^ The indicated conclusions were similar, independent of the age groups, and were also observable among pregnant or postpartum women. It should be emphasized that the indicated associations were stated for the habitual diet, because dietary patterns undergo dynamic changes.^64^ However, the observations for dietary patterns associated with fruit and vegetable intake were confirmed by the observations made for the intake of fruit and vegetables itself (not within the broader pattern), among adults,^27^ children,^65^ and adolescents.^66^

The agreement between the results of the studies analyzing a dietary pattern associated only with fruit and vegetable intake and those analyzing a broader pattern, including within various components the increased intake of fruit and vegetables, may allow us to conclude that fruit and vegetable intake may be responsible for the beneficial impact on mental health. However, the mechanism behind the observed influence is unknown, because a number of potential factors associated with fruit and vegetables could contribute to this effect (eg, they have a high amount of nutrients that have been linked to psychological health),^67^ as discussed by many authors.^68^ Some of these nutrients are vitamins, including vitamin C,^69^ B vitamins,^70^ vitamins A^71^ and K,^72^ as well as minerals, including potassium,^73^ calcium,^74^ magnesium,^75^ and iron.^76^ In addition, other compounds of fruit and vegetables, including polyphenols,^77^ such as flavonoids,^78^ or fiber,^79^ may also influence mental health. As a result, for the time being, it is impossible to indicate a single characteristic of fruit and vegetables to which can be attributed their positive influence on mental health. Moreover, as indicated by Angelino et al,^68^ other potential explanations, directly associated with the assessed pattern, can also be given, namely that the dietary pattern characterized by a high intake of fruit and vegetables is commonly associated with a low energy value and a low intake of unhealthy food products.

Some studies specify that the reverse causality cannot be ruled out as an explanation of the observed association.^58^ Therefore, not only the increased intake of fruit and vegetables may promote better mental health but better mental health may also influence the dietary pattern changes associated with increased consumption of those products. It may be related to the fact that mental health problems are indicated to decrease diet quality and modify eating patterns, as highlighted in the meta-analysis of the dietary intake of individuals with mental illness by Teasdale et al.^80^ However, based on the presented studies, some of them being prospective,^47–49^^,^^52^^,^^54^^,^^55^^,^^57^ the influence of fruit and vegetables on mental health should be concluded. It is in agreement with the observations presented by Angelino et al^68^ in their umbrella review of observational studies indicating a broad positive effect of fruit and vegetable intake on various health outcomes, including depression. Taking this into account, and emphasizing the general health outcomes associated with the intake of fruit and vegetables,^68^ these foods should be recommended as a potential dietary component to improve the general well-being.

## CONCLUSION

A general positive influence was observed for the dietary patterns characterized by high consumption of fruit and vegetables and of fruit or vegetable products. The observed association was not stated in all the included studies; some of them revealed a reverse relationship, but only for the vegetarian/vegan diet. Therefore, when comparing studies that focused on a specific pattern for fruit and vegetables only with studies that focused on broader patterns, fruit and vegetable intake may be confirmed as beneficial for mental health. The studies with the lowest risk of bias indicated that a diet with a large proportion of fruit and vegetables may be associated in women with a reduced risk of anxiety, mental disorders, and depressive symptoms, as well as their consequences, such as suicide. Prospective and/or intervention studies are needed to rule out the reverse causality, but it may be concluded that fruit and vegetables should be included as an element of a properly balanced diet to improve the mental health of women.

## Supplementary Material

nuab007_Supplementary_Data
