# Supplementary material for: Fruit and Vegetable Dietary Patterns and Mental Health in Women: A Systematic Review
Source: Nutr Rev. 2021 May 26;80(6):1357–70. doi: 10.1093/nutrit/nuab007 (PMC9086786; doi:10.1093/nutrit/nuab007)
Supplement: nuab007_Supplementary_Data [file nuab007_supplementary_data.zip › Certificate_No_44412020_TPG.pdf]

Translmed Publishing Group

LANGUAGE EDITING CERTIFICATE

JNL: Translmed No: 44412020 TYPE: L2 DATE: 10/12/2020 EDITOR: VA/TRANSL SPELLING: AmE
Issued: 11/12/2020

Manuscript title:

"Fruit and vegetable dietary patterns and mental health in women: a systematic review"

Authors:
Dominika Guzek, Dominika Głąbska, Barbara Groele, Krystyna Gutkowska

submitted on December 5th was edited for proper English language by an expert language reviewer (native-speaker from the United States). Language verification was performed by employee of Translmed Publishing Group with offices located in Wielun (Poland) and Bedford, NH (USA). If you have any concerns or questions about this document or certification, please contact translmed@translmed.com.
Proofreading & Copyediting: this involved reviewing and editing text for spelling mistakes, typo errors – mainly introduced by mistyping, correcting mistakes in grammar and punctuation, correcting syntax, word usage, capitalization, tenses in the manuscript and improving the flow of text by rewriting, rearranging and rephrasing the sentences for better readability, comprehension and flow, maintaining style, originality and US/UK consistency.

This document should be English-ready for publication; however, the author has the ability to accept or reject suggestions and changes made during the editing process.

38 Hawthorne Dr E106
Bedford, NH, 03110, U.S.A.
Phone: +1 607.319.9069
E-mail: translmed@translmed.com
Website: https://translmed.com/

Member of Council of Science Editors

Oś. Stare Sady 78/27
98-300 Wieluń, Łódzkie
Poland
Phone: +48 (71)710-66-48
