# Supplementary material for: Fruit and Vegetable Dietary Patterns and Mental Health in Women: A Systematic Review
Source: Nutr Rev. 2021 May 26;80(6):1357–70. doi: 10.1093/nutrit/nuab007 (PMC9086786; doi:10.1093/nutrit/nuab007)
Supplement: nuab007_Supplementary_Data [file nuab007_supplementary_data.zip › supplemantary.doc]

**Supplementary Table 1.** The full electronic search strategy applied for PubMed and Web of Science databases.

| **Database** | **The applied full electronic search strategy** |
| --- | --- |
| PubMed | (((fruit[Title/Abstract] OR fruits[Title/Abstract] OR vegetable[Title/Abstract] OR vegetables[Title/Abstract] OR juice[Title/Abstract] OR juices[Title/Abstract])) AND (mental health[Title/Abstract] OR mental disorder[Title/Abstract] OR mental disorders[Title/Abstract] OR psychological distress[Title/Abstract] OR mood disorders[Title/Abstract] OR depression[Title/Abstract] OR anxiety[Title/Abstract] OR suicide[Title/Abstract] OR suicidal[Title/Abstract] OR well-being[Title/Abstract] OR wellbeing[Title/Abstract] OR quality of life[Title/Abstract] OR self-esteem[Title/Abstract] OR self esteem[Title/Abstract] OR self-efficacy[Title/Abstract] OR self efficacy[Title/Abstract] OR resilience[Title/Abstract] OR empowerment[Title/Abstract] OR life skills[Title/Abstract] OR social participation[Title/Abstract] OR mental capital[Title/Abstract] OR emotional[Title/Abstract] OR psychosocial[Title/Abstract] OR psychology[Title/Abstract] OR psychiatry[Title/Abstract])) NOT (animal NOT (animal AND human)[MeSH Terms]) |
| Web of Science | (TS=(fruit OR fruits OR vegetable OR vegetables OR juice OR juices) AND TS=("mental health" OR "mental disorder" OR "mental disorders" OR "psychological distress" OR "mood disorder" OR depression OR anxiety OR suicide OR suicidal OR well-being OR wellbeing OR "quality of life" OR self-esteem OR "self esteem" OR self-efficacy OR "self efficacy" OR resilience OR empowerment OR "life skills" OR "social participation" OR "mental capital" OR emotional OR psychosocial OR psychology OR psychiatry) NOT TS=(animal NOT (animal AND human))) |

**Table 2.** The characteristics of groups of women analyzed in the studies of association between fruit or vegetable dietary pattern and mental health included to the systematic review.

| **Ref.** | **Number of female (total participants)** | **Age for population of women (mean with SD)** | **Inclusion criteria/ Exclusion criteria** |
| --- | --- | --- | --- |
| Boldt et al (2018)^S1^ | 159 (281) | 40.0±11.0 | Inclusion: ≥18 years, successful participation in a running event of either half-marathon or marathon distance in the past two years  Exclusion: Missing data, inconsistent data |
| Forestell and Nezlek (2018)^S2^ | 3,707 (6,422) | 18.96±1.39 | Inclusion: Students in introductory psychology classes who completed the study in partial fulfillment of a course requirement  Exclusion: - |
| Gomes et al (2018)^S3^ | 870 (1,378) | 71.0±8.31 ^a^ | Inclusion: : ≥60 years, living in Pelotas  Exclusion: institutionalized, enteral nutritional therapy |
| Li et al (2018)^S4^ | 571 (1,051) | Not specified | Inclusion: ≥ 65 years, lived at the investigation site for at least 6 months before the survey, no mental illness that could affect normal communication  Exclusion: - |
| Miyake et al (2018)^S5^ | 1,744 (1,744) | 31.2±4.3 | Inclusion: 5th-39th week of pregnancy, living in one of seven prefectures on Kyushu Island or in Okinawa prefecture  Exclusion: Missing data |
| Teo et al (2018)^S6^ | 490 (490) | 18-50 years | Inclusion: 18-50 years, Singaporeans or permanent residents of Singapore, from Chinese, Malay, or Indian ethnic group with homogeneous parental ethnic background, intending to deliver in National University Hospital (NUH) or KK Women’s and Children’s Hospital (KKH), residing in Singapore for the next five years, willing to donate cord blood, cord, and placenta at delivery  Exclusion: Undergoing chemotherapy treatment, using psychotropic drugs, with serious health issues, such as type 1 diabetes mellitus |
| Adjibade et al (2017)^S7^ | 2,031 (3,523) | Not specified | Inclusion: -  Exclusion: Participants with prevalent depressive symptoms at the 1st assessment (defined by CES-D ≥23 for women), participants who reported treatment for depression at baseline |
| Baskin et al (2017)^S8^ | 167 (167) | 30.6±4.2 | Inclusion: ≥18 years, over and between 10 and 16 weeks gestation  Exclusion: - |
| Paskulin et al (2017)^S9^ | 712 (712) | 24.6±6.4 | Inclusion: 16th to 36th gestational weeks, recruited in primary healthcare units, 10 located in Porto Alegre and Bento Gonçalves  Exclusion: - |
| Sakai et al (2017)^S10^ | 7,796 (7,796) | 18 years (young group)  47.9±4.2 (middle-aged group) | Inclusion: Japanese dietetic students with their mothers  Exclusion: Students who answered the questionnaires more than 1 month after the start of the course, subjects living in eastern Japan who participated in the 2011 data collection period, students from one institution at which the response rate was extremely low, missing data |
| Huddy et al (2016)^S11^ | 437 (437) | 32.1±4.3 | Inclusion: Women at 3 months postpartum, first-time mothers, 19-45 years, from the Geelong and Melbourne  Exclusion: Not literate in English, not primiparous, chronic health conditions in the infant that were likely to influence weight, height, activity, or feeding |
| Kim et al (2016)^S12^ | 4196 (8376) | 20–79 years | Inclusion: 20-79 years  Exclusion: Implausible energy intake (<500 or >5000 kcal), missing data |
| Liu et al (2016)^213^ | 906 (906) | 48–65 years | Inclusion: Chinese women, 48-65 years, absence of a period for at least 1 year  Exclusion: History of stroke, gout, or cancers, taking antihypertensive, hypoglycemic, or weight reduction agents, on hormone therapy or vegetarian diet currently or in the preceding 6 months, and long-term antibiotic use; for sub-group within the Whole Soy Study: prehypertension or untreated stage 1 hypertension; for sub-group within the Soy Protein Study: prediabetes or early untreated diabetes |
| Hosseinzadeh et al (2015)^S14^ | Not specified for women (3,846) | Not specified | Inclusion: 20–55 years, working in health centres  Exclusion: Total energy intake of < 800 or > 4200 kcal |
| Vilela et al (2015)^S15^ | 185 (185) | 26.7±5.5 | Inclusion: 20-40 years, 5-13 weeks pregnant  Exclusion: Chronic diseases (except obesity), infectious diseases, during study gave birth to twins, changed their prenatal care health unit, abandoned the prenatal care and follow-up, miscarriage, had a stillbirth, missing data |
| Akbaraly et al (2013)^S16^ | 1,024 (4,053) | Not specified | Inclusion: Civil servants, 35–55 years at baseline  Exclusion: Antidepressant treatment, missing data |
| Chocano-Bedoya et al (2013)^S17^ | 50,605 (50,605) | Not specified | Inclusion: Women , registered nurses  Exclusion: Antidepressant medication use, history of diagnosed depression, severe depressive symptoms on the basis of the 5-item Mental Health Inventory (MHI-5) questionnaires, missing data |
| Ford et al (2013)^S18^ | 6,234 (9,255) | 61.7±13.3 | Inclusion: ≥ 35 years, Seventh Day Adventists  Exclusion: Ethnicities other than Black or White, current smokers, total energy intake > 4500 kcal, missing data |
| Nanri et al (2013)^S19^ | 48,285 (89,037) | Not specified | Inclusion: ≥ 40 years  Exclusion: Extreme total energy intake (≥ mean ± 3 standard deviations according to gender), missing data |
| Rashidkhani et al (2013)^S20^ | 135 (135) | Not specified | Inclusion: 20–45 years  Exclusion: Diseases that limit physical activity, pregnancy and lactation, adherence to a special diet |
| Rienks et al (2013)^S21^ | 7,588 (7,588) for cross-sectional analysis  7,588 (7,588)/6,060 (6,060) for longitudinal analysis | Not specified | Inclusion: 50–55 years  Exclusion: Depressive symptoms, missing data |
| Le Port et al (2012)^S22^ | 3,132 (12,404) | 42.2±4.2 at baseline | Inclusion: 35–50 years, employees of France’s national Gas and Electricity Company (EDF-GDF),  Exclusion: Missing data |
| Chatzi et al (2011)^S23^ | 529 (529) | Not specified | Inclusion: Pregnant, residents of Crete (prefecture of Heraklion)  Exclusion: Gave birth to twins, spontaneous or induced abortions, gave birth to stillborn infants, missing data, diagnosis of depression or other psychiatric disorders before pregnancy, participants with a total energy intake of < 1000 or > 4000 kcal/d |
| Jacka et al (2011)^S24^ | 3,254 (5,731) | Not specified | Inclusion: 46-49 years or 70-74 years  Exclusion: Total energy intake of <2,000 or > 15,000 kJ, missing data |
| Okubo et al (2011)^S25^ | 865 (865) | 29.9±4.0 | Inclusion: Pregnant women, living in Osaka prefecture  Exclusion: Missing data |
| Jacka et al (2010)^S26^ | 1,023 (1,023) | Not specified | Inclusion: Women  Exclusion: Missing data |
| Beydoun et al (2009)^S27^ | 1,003 (1,771) | Not specified | Inclusion: 30-64 years, African Americans and whites, living in Baltimore City  Exclusion: Missing data |
| Muñoz et al (2009)^S28^ | 3,697 (7,145) | 53.8±10.9 | Inclusion: 35-74 years  Exclusion: Total energy intake of <800 or > 4500 kcal, missing data |
| Samieri et al (2008)^S29^ | 1,077 (1,724) | Not specified | Inclusion: ≥65 years, living in Bordeaux  Exclusion: Institutionalized, missing data |
| Yannakoulia et al (2008)^S30^ | 400 (853) | 37±11.0 | Inclusion: Living in the province of Attica  Exclusion: Cold or flu, acute respiratory infection, dental problems or surgery of any kind during the week preceding the study, clinical histories of cardiovascular disease, missing data |

^a^ Data provided by authors on request.

**References**

S1**.** Boldt P, Knechtle B, Nikolaidis P, et al. Quality of life of female and male vegetarian and vegan endurance runners compared to omnivores - results from the NURMI study (step 2). *J Int Soc Sports Nutr.* 2018;15(1):33. doi:10.1186/s12970-018-0237-8.

S2. Forestell CA, Nezlek JB. Vegetarianism, depression, and the five factor model of personality. *Ecol Food Nutr.* 2018;57(3):246‐259. doi:10.1080/03670244.2018.1455675.

S3. Gomes AP, Oliveira Bierhals I, Gonçalves Soares AL, et al. Interrelatioship between Diet Quality and Depressive Symptoms in Elderly. *J Nutr Health Aging.* 2018;22(3):387‐392. doi:10.1007/s12603-017-0963-7.

S4. Li XD, Cao HJ, Xie SY, et al. Adhering to a vegetarian diet may create a greater risk of depressive symptoms in the elderly male Chinese population. *J Affect Disord.* 2019;243:182‐187. doi:10.1016/j.jad.2018.09.033.

S5. Miyake Y, Tanaka K, Okubo H, et al. Dietary patterns and depressive symptoms during pregnancy in Japan: Baseline data from the Kyushu Okinawa Maternal and Child Health Study. *J Affect Disord.* 2018;1(225):552-558. doi: 10.1016/j.jad.2017.08.073.

S6. Teo C, Chia AR, Colega MT, et al. Prospective Associations of Maternal Dietary Patterns and Postpartum Mental Health in a Multi-Ethnic Asian Cohort: The Growing up in Singapore towards Healthy Outcomes (GUSTO) Study. *Nutrients*. 2018;2;10(3):299. doi:10.3390/nu10030299.

S7. Adjibade M, Assmann KE, Andreeva VA, et al. Prospective association between adherence to the Mediterranean diet and risk of depressive symptoms in the French SU.VI.MAX cohort. *Eur J Nutr.* 2018;57(3):1225‐1235. doi:10.1007/s00394-017-1405-3.

S8. Baskin R, Hill B, Jacka FN, et al. Antenatal dietary patterns and depressive symptoms during pregnancy and early post-partum. *Matern Child Nutr.* 2017;13(1):e12218. doi:10.1111/mcn.12218. doi:10.1111/mcn.12218.

S9. Paskulin JTA, Drehmer M, Olinto MT, et al. Association between dietary patterns and mental disorders in pregnant women in Southern Brazil. *Braz J Psychiatry.* 2017;39(3):208‐215. doi:10.1590/1516-4446-2016-2016.

S10. Sakai H, Murakami K, Kobayashi S, et al. Food-based diet quality score in relation to depressive symptoms in young and middle-aged Japanese women. *Br J Nutr.* 2017;117(12):1674-1681. doi:10.1017/S0007114517001581.

S11. Huddy R.L, Torres S.J, Milte C.M, et al. Higher Adherence to the Australian Dietary Guidelines Is Associated with Better Mental Health Status among Australian Adult First-Time Mothers. *J Acad Nutr Diet.* 2016;116(9):1406‐1412. doi:10.1016/j.jand.2016.

S12. Kim WK, Shin D, Song WO. Are Dietary Patterns Associated with Depression in U.S. Adults? *J Med Food.* 2016;19(11):1074‐1084. doi:10.1089/jmf.2016.0043.

S13. Liu ZM, Ho SC, Xie YJ et al. Associations between dietary patterns and psychological factors: a cross-sectional study among Chinese postmenopausal women. *Menopause.* 2016;23(12):1294‐1302. doi:10.1097/GME.0000000000000701.

S14. Hosseinzadeh M, Vafa M, Esmaillzadeh A, et al. Empirically derived dietary patterns in relation to psychological disorders. *Public Health Nutr.* 2016;19(2):204‐217. doi:10.1017/S136898001500172X.

S15. Vilela AA, Pinto Tde J, Rebelo F, et al. Association of Prepregnancy Dietary Patterns and Anxiety Symptoms from Midpregnancy to Early Postpartum in a Prospective Cohort of Brazilian Women. *J Acad Nutr Diet.* 2015;115(10):1626‐1635. doi:10.1016/j.jand.2015.01.007.

S16. Akbaraly TN, Sabia S, Shipley MJ, et al. Adherence to healthy dietary guidelines and future depressive symptoms: evidence for sex differentials in the Whitehall II study. *Am J Clin Nutr.* 2013;97(2):419-427. doi: 10.3945/ajcn.112.041582.

S17. Chocano-Bedoya PO, O'Reilly EJ, Lucas M, et al. Prospective study on long-term dietary patterns and incident depression in middle-aged and older women. *Am J Clin Nutr.* 2013;98(3):813‐820. doi:10.3945/ajcn.112.052761.

S18. Ford PA, Jaceldo-Siegl K, Lee JW, et al. Intake of Mediterranean foods associated with positive affect and low negative affect. *J Psychosom Res.* 2013;74(2):142-148. doi: 10.1016/j.jpsychores.2012.11.002.

S19. Nanri A, Mizoue T, Poudel-Tandukar K, Noda M, et al. Japan Public Health Center-based Prospective Study Group. Dietary patterns and suicide in Japanese adults: the Japan Public Health Center-based Prospective Study. *Br J Psychiatry.* 2013;203(6):422‐427. doi:10.1192/bjp.bp.112.114793.

S20. Rashidkhani B, Pourghassem Gargari B, Ranjbar F, et al. Dietary patterns and anthropometric indices among Iranian women with major depressive disorder. *Psychiatry Res.* 2013;210(1):115‐120. doi:10.1016/j.psychres.2013.05.022.

S21. Rienks J, Dobson AJ, Mishra GD. Mediterranean dietary pattern and prevalence and incidence of depressive symptoms in mid-aged women: results from a large community-based prospective study. *Eur J Clin Nutr.* 2013;67(1):75‐82. doi:10.1038/ejcn.2012.193.

S22. Le Port A, Gueguen A, Kesse-Guyot E, et al. Association between dietary patterns and depressive symptoms over time: a 10-year follow-up study of the GAZEL cohort. *PLoS One.* 2012;7(12):e51593. doi:10.1371/journal.pone.0051593.

S23. Chatzi L, Melaki V, Sarri K, et al. Dietary patterns during pregnancy and the risk of postpartum depression: the mother-child 'Rhea' cohort in Crete, Greece. P*ublic Health Nutr.* 2011;14(9):1663‐1670. doi:10.1017/S1368980010003629.

S24. Jacka FN, Mykletun A, Berk M, et al. The association between habitual diet quality and the common mental disorders in community-dwelling adults: the Hordaland Health study. *Psychosom Med.* 2011;73(6):483‐490. doi:10.1097/PSY.0b013e318222831a.

S25. Okubo H, Miyake Y, Sasaki S, et al. Osaka Maternal and Child Health Study Group. Dietary patterns during pregnancy and the risk of postpartum depression in Japan: the Osaka Maternal and Child Health Study. *Br J Nutr.* 2011;105(8):1251‐1257. doi:10.1017/S0007114510004782.

S26. Jacka FN, Pasco JA, Mykletun A, et al. Association of Western and traditional diets with depression and anxiety in women. *Am J Psychiatry.* 2010;167(3):305‐311. doi:10.1176/appi.ajp.2009.09060881.

S27. Beydoun MA, Kuczmarski MT, Mason MA, et al. Role of depressive symptoms in explaining socioeconomic status disparities in dietary quality and central adiposity among US adults: a structural equation modeling approach. *Am J Clin Nutr.* 2009;90(4):1084‐1095. doi:10.3945/ajcn.2009.27782.

S28. Muñoz MA, Fíto M, Marrugat J, et al. Adherence to the Mediterranean diet is associated with better mental and physical health. *Br J Nutr.* 2009;101(12):1821‐1827. doi:10.1017/S0007114508143598.

S29. Samieri C, Jutand MA, Féart C, et al. Dietary patterns derived by hybrid clustering method in older people: association with cognition, mood, and self-rated health. *J Am Diet Assoc.* 2008;108(9):1461‐1471. doi:10.1016/j.jada.2008.06.437.

S30. Yannakoulia M, Panagiotakos DB, Pitsavos C, et al. Eating habits in relations to anxiety symptoms among apparently healthy adults. A pattern analysis from the ATTICA Study. *Appetite.* 2008;1(3):519‐525. doi:10.1016/j.appet.2008.04.002.

**Table 3.** The detailed description of compared dietary patterns in the studies included to the systematic review.

| **Ref.** | **Assessment** | **Fruit and vegetable dietary pattern** | **Other dietary pattern** |
| --- | --- | --- | --- |
| Boldt et al (2018)^S1^ | Question about the preferred diet | Vegetarian (no meat)/vegan (no products from animal sources, such as meat, fish, milk and dairy products, eggs and honey) diet | Omnivorous diet (Western diet, no dietary restrictions) |
| Forestell and Nezlek (2018)^S2^ | General Eating Habits Scale | Vegan (fruits, vegetables, and grains but no animal or seafood products); Lacto-vegetarian (fruits, vegetables, grains, and dairy products, but no other animal or seafood products); Lacto-ovo-vegetarian (fruits, vegetables, grains, dairy products, and eggs, but no other animal or seafood products); Pesco-vegetarian (fruits, vegetables, grains, dairy products, eggs, and seafood, but no other animal products); Semi-vegetarian (fruits, vegetables, grains, dairy products, eggs, seafood, and chicken but no red meat) | Occasional omnivore (occasionally red meat, white meat, seafood, eggs, dairy products, fruits, vegetables, and grains); Omnivore (regularly most meats, seafood, eggs, dairy products, fruits, vegetables, and grains) |
| Gomes et al (2018)^S3^ | Elderly Dietary Quality Index (EDQ-I) | “Healthy” diet based on EDQ-I for higher consumption of “healthy” groups: rice and beans, whole grain, fruit, vegetables, milk and dairy products and beef, fish, chicken or eggs. | “Unhealthy” diet based on EDQ-I for higher consumption of “unhealthy” groups: sweets, soft drinks and artificial juices (defined as beverages)*, fried food, canned food and sausages, ready-to-eat and frozen food, fast food |
| Li et al (2018)^S4^ | Food frequency questionnaire | “Vegetable-based” diet (eating vegetables frequently) | “Meat-based” diet (eating meat frequently), “Balanced” pattern (meat and vegetables with the same frequency) |
| Miyake et al (2018)^S5^ | Semi-quantitative, comprehensive diet history questionnaire (DHQ) with 150 food items | “Healthy” dietary pattern (high intake of green and yellow vegetables, other vegetables, mushrooms, pulses, seaweed, potatoes, fish, sea products, miso soup, sugar, shellfish) | “Japanese” dietary pattern (high intake of rice and miso soup), “Western” dietary pattern (high intake of beef and pork, processed meat, vegetable oil, chicken, eggs, shellfish, and salt-containing seasonings) |
| Teo et al (2018)^S6^ | 3-day dietary records | “Soup, Vegetables and Fruits” diet (high consumption of assorted soup (vegetables, meat, fish, seafood, and noodles), vegetables, fish (non-fried), and fresh fruits, and lower intakes of sweet spreads and milk-based drinks) | “Traditional Chinese Confinement” diet (high consumption of traditional dried fruits, herbal tea, rhizomes, Chinese herbs, and foods cooked with wine, alcohol, or vinegar), “Traditional Indian Confinement” diet (high consumption of ethnic bread, Indian herbs, whole milk, seed herbs, and butter/ghee), “Eat-Out” diet (high consumption of deep-fried/mashed potato, sweetened and cordial drinks, ice-cream, chips/crisps, deep-fried dimsum, and local savoury snacks) |
| Adjibade et al (2017)^S7^ | Repeated 24-h dietary records | Adherence to Mediterranean diet defined based on relative Mediterranean diet score (rMED) for high consumption of the “desirable” components (grains, vegetables (excluding potatoes), legumes, fruits and nuts, fresh fish, olive oil, and moderate alcohol consumption) | No adherence to Mediterranean diet defined based on relative Mediterranean diet score (rMED) for high consumption of the “undesirable” components (meat, dairy products) |
| Baskin et al (2017)^S8^ | Cancer Council Victoria Food Frequency Questionnaire | “Healthy” dietary pattern (including vegetables, fruit, eggs, nuts, fish and seafood, water, whole grains and tea) | “Unhealthy” dietary pattern (including condiments, sweets and desserts, refined grains, high-energy drinks, fast foods, hot chips, high-fat dairy, fruit juice and red meats) |
| Paskulin et al (2017)^S9^ | Food frequency questionnaire with 88 food items | “Varied” dietary pattern (including grains, cereals, and tubers, breads, cakes, and cookies, fruits, and vegetables) | “Restricted” dietary pattern (including easy-to-prepare foods, including industrialized foods, consistent with contemporary eating patterns that feature limited food variability - fast food, sweets, desserts, high-fat products, and sweetened beverages, “Common-Brazilian” dietary pattern (including rice or noodles, French rolls, beans, boneless beef/chicken or eggs, coffee with sugar, margarine, and artificial juices (defined as beverages)*) |
| Sakai et al (2017)^S10^ | Comprehensive Diet History Questionnaire (DHQ) | Adherence to healthy diet defined based on diet quality score calculated based on the intake of components recommended in the Japanese Food Guide Spinning Top (grain dishes, vegetable dishes, fish and meat dishes, milk, fruits, snacks, confection and beverages), and sodium from seasonings | No adherence to healthy diet defined based on diet quality score calculated based on the intake of components recommended in the Japanese Food Guide Spinning Top, and sodium from seasonings |
| Huddy et al (2016)^S11^ | Cancer Council Victoria Food Frequency Questionnaire (Dietary Questionnaire  for Epidemiological Studies version 3.1) | Adherence to the 2013 Australian Dietary Guidelines assessed using the Dietary Guideline Index (including: diet variety, vegetables, fruit, grains and cereals, meat and alternatives, dairy and alternatives, discretionary foods, fat and sugar, and alcohol) | No adherence to the 2013 Australian Dietary  Guidelines assessed using the Dietary Guideline Index |
| Kim et al (2016)^S12^ | 24-h dietary recall | ‘‘Healthy’’ dietary pattern (high in whole grain, dark-green, orange, and other vegetables, tomatoes, fruits, fish and shellfish, and nuts and seeds) | ‘‘Western’’ dietary pattern (high in on nonwhole/refined grain, white potatoes, tomatoes, cheese, meat, frankfurters, sausage and luncheon meats, eggs, discretionary oil, discretionary solid fat, and added sugars) |
| Liu et al (2016)^213^ | Food frequency questionnaire with 85 food items | “Whole plant foods” dietary pattern (high intake of whole grains, vegetables, and fruits) | “Processed foods” dietary pattern (high intake of refined grains, preserved/salted foods, fat/semi-lean meat, fried foods, and sweets), “Animal food” dietary pattern (high intake of fish, lean meat, and milk products) |
| Hosseinzadeh et al (2015)^S14^ | Dish-based semiquantitative food frequency questionnaire with 106 food items (DS-FFQ) | “Lacto-vegetarian” dietary pattern (high in non-flatulent vegetables, tomato, citrus fruits, flatulent vegetables, fruits and low-fat dairy products) | “Fast food” dietary pattern (high in French fries, vegetable oils, meat, pepper, salt, onions, soya and eggs), “Traditional” dietary pattern (high in vegetable oils, meat, salt, legumes, non-flatulent vegetables, poultry, hydrogenated vegetable oils, dried fruits, fish and organ meats), “Western” dietary pattern (high in snacks, high-fat dairy products, chocolate, carbonated drinks, sweets and desserts) |
| Vilela et al (2015)^S15^ | Food frequency questionnaire with 82 food items | “Healthy” prepregnancy dietary pattern (roots and tubers, green vegetables and legumes, fruits and fruit juice, dairy products, fish, tea, pasta, cakes and cookies-crackers, and candies) | “Common-Brazilian” prepregnancy dietary pattern (rice, beans, meats and eggs, and vegetable spices), “processed” prepregnancy dietary pattern (bread, sugar, fat, fast food and snacks, soft drinks, and sausages and deli meats), |
| Akbaraly et al (2013)^S16^ | Food frequency questionnaire with 127 food items | Adherence to healthy diet defined based on the Alternative Healthy Eating Index (AHEI) score (calculated based on intake of fruit, vegetable, ratio of white meat to red meat; trans fat, ratio of PUFA to SFA, total fiber, nuts and soy, alcohol, long-term multivitamin use) | No adherence to healthy diet defined based on the Alternative Healthy Eating Index (AHEI) score |
| Chocano-Bedoya et al (2013)^S17^ | Food frequency questionnaires with 61 and 131 food items | “Prudent” dietary pattern (high in fruit, vegetables, fish, whole-grain products, low-fat dairy) | ‘‘Western’’ dietary pattern (high in red and processed meats, French fries, desserts, high-fat dairy, refined grains) |
| Ford et al (2013)^S18^ | Food frequency questionnaire with over 200 food items | Diet including “Mediterranean foods” (non-starchy fresh vegetables, fresh fruit, certain dairy products, olive oil, nuts, fish, and legumes, excluding soy) | Diet including “Western foods” (red meats, processed meats, sweets/desserts, soda, and fast foods) |
| Nanri et al (2013)^S19^ | Food frequency questionnaire with 147 food items | “Japanese” dietary pattern (high intake of vegetables, fruits, soy products, potatoes, seaweed, mushrooms, fish) | “Westernized Japanese” dietary pattern (high intake of meat, processed meat, bread, dairy products, coffee, black tea, soft drink, dressing, sauce, mayonnaise), “traditional Japanese” dietary pattern (high intake of salmon, salty fish, oily fish, seafoods other than fish, pickles) |
| Rashidkhani et al (2013)^S20^ | Food frequency questionnaire with 125 food items | “Healthy” dietary pattern (high intake of fish, poultry, low fat dairy, high fat dairy, coffee, fruits and nuts, fruit juices, vegetables, legumes, olive) | “Unhealthy” dietary pattern (high intake of processed meats, red meat, tea, fried potatoes, whole grains, refined grains, snacks, cookies, oils, sugar, soft drinks) |
| Rienks et al (2013)^S21^ | Food frequency questionnaire with 101 food items | “Cooked vegetables” dietary pattern (including cauliflower, cabbage, Brussels sprouts, broccoli and green beans), “fruit” dietary pattern (including strawberries, pineapple, melon, apricots and mango), “Mediterranean style” dietary pattern (including garlic, peppers, mushrooms, salad greens, pasta and red wine) | “Meat and processed meat” dietary pattern (including pork, bacon, sausages and lamb), “dairy” dietary pattern (including cream cheese, low-fat cheese, yoghurt and skim milk), “high fat and sugar” dietary pattern (including sweet biscuits, cakes, jam, meat pies and chocolate) |
| Le Port et al (2012)^S22^ | Food frequency questionnaire with 35 food items | “Healthy” dietary pattern (high intake of cooked vegetables, raw vegetables, fruits, oil), “traditional” dietary pattern (high intake of fish and fruits) | “Low-fat” dietary pattern (high intake of fat-free dairy products, fat-free cheese, margarine, fat-free dishes, diet sodas, sugar free candies, sweetener), “animal protein” dietary pattern (high intake of meat, poultry, processed meat, eggs), “dessert” dietary pattern (high intake of processed meat, dairy products, cheese, dessert, pastries, sweetener) |
| Chatzi et al (2011)^S23^ | Food frequency questionnaire (Rhea FFQ) with 250 food items | “Health conscious” dietary pattern (vegetables, fruit, nuts, pulses, fish and seafood, olive oil, dairy products) | “Western” dietary pattern (meat and meat products, potatoes, sugar and sweets, cereals, fats except olive oil, salty snacks, eggs, beverages, sauces) |
| Jacka et al (2011)^S24^ | Food frequency questionnaire with 169 food items | “Healthy” dietary pattern (high intake of vegetables, salads, fruits, rice, pasta, cereals, fish, wine, nonprocessed meats), “traditional (Norwegian)” dietary pattern (high intake of fish and shellfish, potatoes, fruits, vegetables, butter and margarine, milk and yogurt, bread, pasta, rice, meat, meat spreads, legumes, and eggs) | “Western” dietary pattern (high intake of meat and liver, processed meats, pizza, salty snacks, chocolates, sugar and sweets, soft drinks, margarine, mayonnaise and other dressings, French fries, beer, coffee, cake, and ice cream) |
| Okubo et al (2011)^S25^ | Comprehensive Diet History Questionnaire (DHQ) with 150 food items | “Healthy” dietary pattern (green and yellow vegetables, seaweeds, white vegetables, potatoes, fish, fruits, shellfish and sea products) | “Western” dietary pattern (vegetable oil, beef and pork, salt-containing seasonings, processed meat, chicken, eggs), “Japanese” dietary pattern (rice, miso soup, sea products, fish and pickled vegetables) |
| Jacka et al (2010)^S26^ | Food frequency questionnaire (Cancer Council Vic­toria dietary questionnaire) with 80 food items | 1) “Traditional” dietary pattern (high intake of vegetables, fruit, beef, lamb, fish, and whole-grain foods), “Modern” dietary pattern (high intake of fruits, salads, fish, tofu, beans, nuts, yogurt, red wine); 2) Adherence to healthy diet defined based on diet quality score calculated based on the Australian national guidelines (including at least 2 servings of fruit a day, at least 4 servings of vegetables per day, red meat consumption 1 to 5 times per week, use of low-fat dairy products, and use of high-fiber, whole-grain, rye, or multigrain breads) | 1) “Western“ dietary pattern (high intake of meat pies, processed meats, pizza, chips, hamburgers, white bread, sugar, flavored milk drinks, beer)  2) No adherence to healthy diet defined based on diet quality score calculated based on the Australian national guidelines |
| Beydoun et al (2009)^S27^ | Two 24-h recall | Adherence to healthy diet defined based on 2005 USDA Healthy Eating Index (HEI) – including 12 components: total fruit (includes 100% juice), whole fruit (not juice), total vegetables, dark green and orange vegetables and legumes, total grains, whole grains, milk, meat and bean, oils, saturated fat, sodium, calories from solid fat, alcohol, and added sugar (SoFAAS) | No adherence to healthy diet defined based on 2005 USDA Healthy Eating Index (HEI) |
| Muñoz et al (2009)^S28^ | Food frequency questionnaire with 165 food items | Adherence to Mediterranean diet defined based on Mediterranean diet score (MDS) – calculated based on the intake of cereals, vegetables, fruits, legumes, nuts, fish, dairy products, olive oil, meat and red wine | No adherence to healthy diet defined based on Mediterranean diet score (MDS) |
| Samieri et al (2008)^S29^ | Food frequency questionnaire | „Healthy” dietary pattern cluster (high in fruit and vegetable) | “Small eaters” dietary pattern cluster (did not represent a particular food group), “biscuits and snacking” dietary pattern cluster, “charcuterie and starchy foods” dietary pattern cluster, “pizza, sandwich” dietary pattern cluster |
| Yannakoulia et al (2008)^S30^ | EPIC-Greek semi-quantitative food frequency questionnaire with 156 food items | “Healthful” dietary pattern (high intake of vegetables, cereals, fish, dairy), “Vegetarian” dietary pattern (high intake of vegetables, fruits, nuts, potatoes) | “Sweets and soft drinks” dietary pattern, ”low-fat/low-sugar products" dietary pattern (low-fat dairy products, poultry, light soft drinks), “Western-type dietary” dietary pattern (high intake of potatoes, red meat, coffee) |

**References**

S1**.** Boldt P, Knechtle B, Nikolaidis P, et al. Quality of life of female and male vegetarian and vegan endurance runners compared to omnivores - results from the NURMI study (step 2). *J Int Soc Sports Nutr.* 2018;15(1):33. doi:10.1186/s12970-018-0237-8.

S2. Forestell CA, Nezlek JB. Vegetarianism, depression, and the five factor model of personality. *Ecol Food Nutr.* 2018;57(3):246‐259. doi:10.1080/03670244.2018.1455675.

S3. Gomes AP, Oliveira Bierhals I, Gonçalves Soares AL, et al. Interrelatioship between Diet Quality and Depressive Symptoms in Elderly. *J Nutr Health Aging.* 2018;22(3):387‐392. doi:10.1007/s12603-017-0963-7.

S4. Li XD, Cao HJ, Xie SY, et al. Adhering to a vegetarian diet may create a greater risk of depressive symptoms in the elderly male Chinese population. *J Affect Disord.* 2019;243:182‐187. doi:10.1016/j.jad.2018.09.033.

S5. Miyake Y, Tanaka K, Okubo H, et al. Dietary patterns and depressive symptoms during pregnancy in Japan: Baseline data from the Kyushu Okinawa Maternal and Child Health Study. *J Affect Disord.* 2018;1(225):552-558. doi: 10.1016/j.jad.2017.08.073.

S6. Teo C, Chia AR, Colega MT, et al. Prospective Associations of Maternal Dietary Patterns and Postpartum Mental Health in a Multi-Ethnic Asian Cohort: The Growing up in Singapore towards Healthy Outcomes (GUSTO) Study. *Nutrients*. 2018;2;10(3):299. doi:10.3390/nu10030299.

S7. Adjibade M, Assmann KE, Andreeva VA, et al. Prospective association between adherence to the Mediterranean diet and risk of depressive symptoms in the French SU.VI.MAX cohort. *Eur J Nutr.* 2018;57(3):1225‐1235. doi:10.1007/s00394-017-1405-3.

S8. Baskin R, Hill B, Jacka FN, et al. Antenatal dietary patterns and depressive symptoms during pregnancy and early post-partum. *Matern Child Nutr.* 2017;13(1):e12218. doi:10.1111/mcn.12218. doi:10.1111/mcn.12218.

S9. Paskulin JTA, Drehmer M, Olinto MT, et al. Association between dietary patterns and mental disorders in pregnant women in Southern Brazil. *Braz J Psychiatry.* 2017;39(3):208‐215. doi:10.1590/1516-4446-2016-2016.

S10. Sakai H, Murakami K, Kobayashi S, et al. Food-based diet quality score in relation to depressive symptoms in young and middle-aged Japanese women. *Br J Nutr.* 2017;117(12):1674-1681. doi:10.1017/S0007114517001581.

S11. Huddy R.L, Torres S.J, Milte C.M, et al. Higher Adherence to the Australian Dietary Guidelines Is Associated with Better Mental Health Status among Australian Adult First-Time Mothers. *J Acad Nutr Diet.* 2016;116(9):1406‐1412. doi:10.1016/j.jand.2016.

S12. Kim WK, Shin D, Song WO. Are Dietary Patterns Associated with Depression in U.S. Adults? *J Med Food.* 2016;19(11):1074‐1084. doi:10.1089/jmf.2016.0043.

S13. Liu ZM, Ho SC, Xie YJ et al. Associations between dietary patterns and psychological factors: a cross-sectional study among Chinese postmenopausal women. *Menopause.* 2016;23(12):1294‐1302. doi:10.1097/GME.0000000000000701.

S14. Hosseinzadeh M, Vafa M, Esmaillzadeh A, et al. Empirically derived dietary patterns in relation to psychological disorders. *Public Health Nutr.* 2016;19(2):204‐217. doi:10.1017/S136898001500172X.

S15. Vilela AA, Pinto Tde J, Rebelo F, et al. Association of Prepregnancy Dietary Patterns and Anxiety Symptoms from Midpregnancy to Early Postpartum in a Prospective Cohort of Brazilian Women. *J Acad Nutr Diet.* 2015;115(10):1626‐1635. doi:10.1016/j.jand.2015.01.007.

S16. Akbaraly TN, Sabia S, Shipley MJ, et al. Adherence to healthy dietary guidelines and future depressive symptoms: evidence for sex differentials in the Whitehall II study. *Am J Clin Nutr.* 2013;97(2):419-427. doi: 10.3945/ajcn.112.041582.

S17. Chocano-Bedoya PO, O'Reilly EJ, Lucas M, et al. Prospective study on long-term dietary patterns and incident depression in middle-aged and older women. *Am J Clin Nutr.* 2013;98(3):813‐820. doi:10.3945/ajcn.112.052761.

S18. Ford PA, Jaceldo-Siegl K, Lee JW, et al. Intake of Mediterranean foods associated with positive affect and low negative affect. *J Psychosom Res.* 2013;74(2):142-148. doi: 10.1016/j.jpsychores.2012.11.002.

S19. Nanri A, Mizoue T, Poudel-Tandukar K, Noda M, et al. Japan Public Health Center-based Prospective Study Group. Dietary patterns and suicide in Japanese adults: the Japan Public Health Center-based Prospective Study. *Br J Psychiatry.* 2013;203(6):422‐427. doi:10.1192/bjp.bp.112.114793.

S20. Rashidkhani B, Pourghassem Gargari B, Ranjbar F, et al. Dietary patterns and anthropometric indices among Iranian women with major depressive disorder. *Psychiatry Res.* 2013;210(1):115‐120. doi:10.1016/j.psychres.2013.05.022.

S21. Rienks J, Dobson AJ, Mishra GD. Mediterranean dietary pattern and prevalence and incidence of depressive symptoms in mid-aged women: results from a large community-based prospective study. *Eur J Clin Nutr.* 2013;67(1):75‐82. doi:10.1038/ejcn.2012.193.

S22. Le Port A, Gueguen A, Kesse-Guyot E, et al. Association between dietary patterns and depressive symptoms over time: a 10-year follow-up study of the GAZEL cohort. *PLoS One.* 2012;7(12):e51593. doi:10.1371/journal.pone.0051593.

S23. Chatzi L, Melaki V, Sarri K, et al. Dietary patterns during pregnancy and the risk of postpartum depression: the mother-child 'Rhea' cohort in Crete, Greece. P*ublic Health Nutr.* 2011;14(9):1663‐1670. doi:10.1017/S1368980010003629.

S24. Jacka FN, Mykletun A, Berk M, et al. The association between habitual diet quality and the common mental disorders in community-dwelling adults: the Hordaland Health study. *Psychosom Med.* 2011;73(6):483‐490. doi:10.1097/PSY.0b013e318222831a.

S25. Okubo H, Miyake Y, Sasaki S, et al. Osaka Maternal and Child Health Study Group. Dietary patterns during pregnancy and the risk of postpartum depression in Japan: the Osaka Maternal and Child Health Study. *Br J Nutr.* 2011;105(8):1251‐1257. doi:10.1017/S0007114510004782.

S26. Jacka FN, Pasco JA, Mykletun A, et al. Association of Western and traditional diets with depression and anxiety in women. *Am J Psychiatry.* 2010;167(3):305‐311. doi:10.1176/appi.ajp.2009.09060881.

S27. Beydoun MA, Kuczmarski MT, Mason MA, et al. Role of depressive symptoms in explaining socioeconomic status disparities in dietary quality and central adiposity among US adults: a structural equation modeling approach. *Am J Clin Nutr.* 2009;90(4):1084‐1095. doi:10.3945/ajcn.2009.27782.

S28. Muñoz MA, Fíto M, Marrugat J, et al. Adherence to the Mediterranean diet is associated with better mental and physical health. *Br J Nutr.* 2009;101(12):1821‐1827. doi:10.1017/S0007114508143598.

S29. Samieri C, Jutand MA, Féart C, et al. Dietary patterns derived by hybrid clustering method in older people: association with cognition, mood, and self-rated health. *J Am Diet Assoc.* 2008;108(9):1461‐1471. doi:10.1016/j.jada.2008.06.437.

S30. Yannakoulia M, Panagiotakos DB, Pitsavos C, et al. Eating habits in relations to anxiety symptoms among apparently healthy adults. A pattern analysis from the ATTICA Study. *Appetite.* 2008;1(3):519‐525. doi:10.1016/j.appet.2008.04.002.

**Table 4.** The observations and conclusions defined for the studies of association between fruit or vegetable dietary pattern and mental health included to the systematic review.

| **Ref.** | **Observation** | **Conclusion** |
| --- | --- | --- |
| Boldt et al (2018)^S1^ | Adhering to an omnivorous diet affected environment scores of WHOQOL-BREF for women, but not psychological well-being. | Endurance runners had a high quality of life regardless of the race distance or diet choice. The findings support the notion that adhering to a vegetarian or vegan diet can be an appropriate and equal alternative to an omnivorous diet. |
| Forestell and Nezlek (2018)^S2^ | There was a significant effect of General Eating Habits (GEH) for women, F(2, 3150) = 4.08, p = 0.017, such that semi-vegetarians were significantly more depressed than omnivores (p < 0.047); however vegetarians did not differ from either semi-vegetarians (p = 0.765) or omnivores (p = 0.136). | Although vegetarians and semi-vegetarians were more open to new experiences, they were more neurotic and depressed than omnivores. |
| Gomes et al (2018)^S3^ | Elderly with low-quality diet were more likely to experience depressive symptoms, and the association was almost twice higher in males than in females (women OR = 2.1, 95% CI: 1.4–3.3). | The choice of a low-quality diet was associated to a higher risk of depressive symptoms in elderly, and vice-versa. |
| Li et al (2018)^S4^ | The elderly participants who had a vegetable-based diet had the highest geriatric depression scale (GDS) scores of 8.78±6.894 (p = 0.001) and the highest rate of depression (32.9%, p = 0.003). After adjustment for the potential confounders, it was not observed in the case in women. | Vegetarian diets may pose a greater risk of depressive symptoms among the elderly Chinese population, especially elderly men. |
| Miyake et al (2018)^S5^ | The healthy and Japanese patterns were independently inversely associated with depressive symptoms during pregnancy: the adjusted prevalence ratios (95% confidence intervals, p for trend) between extreme quartiles were 0.56 (0.43–0.73, p <0.0001) and 0.72 (0.55–0.94, p = 0.008), respectively. No association was observed between the Western pattern and depressive symptoms during pregnancy. | The healthy and Japanese dietary patterns may be inversely associated with depressive symptoms during pregnancy. |
| Teo et al (2018)^S6^ | The “Traditional-Indian-Confinement” diet was associated with less postpartum depression (PPD) symptoms [β (95% CI) -0.62 (-1.16, -0.09) Edinburgh Postnatal Depression Scale (EPDS) score per SD increase in diet score] and a non-significant trend with reduced probable PPD (EPDS scores ≥ 13) [OR (95% CI) 0.56 (0.31, 1.01)]. The “Soup-Vegetables-Fruits” diet was associated with less postpartum anxiety (PPA) symptoms [β (95% CI) -1.49 (-2.56, -0.42) State-Trait Anxiety Inventory -state score]. No associations were observed for other dietary patterns. | Adherence to the Traditional-Indian-Confinement diet that is characterized by intake of herbs and legumes, and Soup-Vegetables-Fruits diet high in fruits, vegetables and fish during the postpartum period were associated with less postpartum depression and postpartum anxiety symptoms, respectively. |
| Adjibade et al (2017)^S7^ | After adjustment for a wide range of potential confounders, adherence to the rMED score (continuous variable) in women was not associated with incident depressive symptoms. | Higher adherence to the Mediterranean Diet at midlife was associated with a lower risk of incident depressive symptoms, particularly in men. |
| Baskin et al (2017)^S8^ | “Unhealthy” dietary pattern diet was associated with increased depressive symptoms at 32 weeks gestation | It is the initial evidence in support of an association between poor diet quality and increased depressive symptoms. |
| Paskulin et al (2017)^S9^ | Women with a common-Brazilian dietary pattern had higher prevalence of major depressive disorder compared to those with a varied consumption pattern (PR 1.43, 95% CI 1.01–2.02). | Low consumption of fruits and beans and intake of the common-Brazilian dietary pattern during pregnancy were associated with higher prevalence of mental disorders. |
| Sakai et al (2017)^S10^ | After adjustment for potential confounders, OR for depressive symptoms in the highest v. lowest quintiles of the diet quality score was 0.65 (95% CI 0.50–0.84) in young women (p = 0.0005). In middle-aged women, the corresponding value was 0·59 (95% CI 0.45–0.78) (p < 0.0001). Analyses where the diet quality and CES-D scores were treated as continuous variables also showed inverse associations. | A higher diet quality score was associated with a lower prevalence of depressive symptoms in young and middle-aged Japanese women. |
| Huddy et al (2016)^S11^ | Better diet quality, as indicated by a higher score on the Dietary Guideline Index, was associated with lower depressive symptoms after adjusting for relevant covariates (β=-0.034; 95% CI -0.056 to-0.012). | Adherence to the Australian Dietary Guidelines was associated with better mental health status among first-time mothers. |
| Kim et al (2016)^S12^ | The ‘‘Western’’ dietary pattern was not significantly associated with depression in women. The ‘‘Healthy’’ dietary pattern scores were inversely associated with the PHQ-9 depression scores and odd ratios (ORs) of depression after adjustment for covariates in women. The OR of depression in women with the highest quintile of ‘‘Healthy’’ dietary pattern scores was 0.60 (95% confidence interval [CI]: 0.42–0.85, p < 0.001) compared to the lowest quintile as a reference. | Findings suggest the close association of consuming ‘‘Healthy’’ diet with a lower risk of depression only in women. |
| Liu et al (2016)^213^ | Multivariable linear regression analyses indicated that whole plant food intake was negatively associated with depression score (p = 0.030). Processed food intake was positively associated with perceived stress (p = 0.025) and depression (p = 0.073), and negatively associated with scores of self-esteem (p = 0.046). The highest tertile of processed foods score was associated with 79.3% increased risk of depression (p for trend = 0.006), whereas the highest tertile of whole plant food score was associated with 26% reduction of depression (p for trend = 0.023) relative to the lowest tertile. | Dietary patterns featuring a low intake of processed foods and/or a high intake of whole plant foods were associated with a reduced risk of depression and perceived stress. |
| Hosseinzadeh et al (2015)^S14^ | After adjustment for potential confounders, greater adherence to the lactovegetarian dietary pattern was protectively associated with depression in women (OR 0.65; 95 % CI 0.46–0.91). Normal-weight participants in the top quintile of this dietary pattern tended to have decreased odds of anxiety compared with those in the bottom quintile (OR 0.61; 95 % CI 0.38–1.00). In addition, the traditional dietary pattern was associated with increased odds of depression (OR 1.42; 95 % CI 1.01–1.99) and anxiety (OR 1.56; 95 % CI 1.00–2.42) in women. Normal weight participants in the highest quintile of the traditional dietary pattern had greater odds for anxiety (OR 1.89; 95 % CI 1.12–3.08) compared with those in the lowest quintile. The Western dietary pattern was associated with increased odds of anxiety in normal-weight participants (OR 2.05; 95 % CI 1.22–3.46). There was a significant increasing trend in the odds of psychological distress across increasing quintiles of the fast food dietary pattern in women (p trend 0 = 0.02). | Recommendation to increase the intake of fruits, citrus fruits, vegetables, tomato and low-fat dairy products and to reduce the intakes of snacks, high-fat dairy products, chocolate, carbonated drinks, sweets and desserts might be associated with lower chance of psychological disorders. |
| Vilela et al (2015)^S15^ | The common-Brazilian pattern (β=-1.200, 95% CI -2.220 to -0.181; p = 0.021), and the healthy pattern (β=-1.290, 95% CI -2.438 to -0.134; p = 0.029) were negatively associated with prospective changes in anxiety symptoms. | High adherence to the common-Brazilian or healthy patterns was negatively associated with higher anxiety symptom scores from mid-pregnancy to early postpartum in this group of Brazilian women. |
| Akbaraly et al (2013)^S16^ | After adjustment for potential confounders, the Alternative Healthy Eating Index (AHEI) score was inversely associated with recurrent depressive symptoms in a dose-response fashion in women (p trend = 0.001; for 1 SD in AHEI score; OR 0.59; 95% CI: 0.47–0.75).Women who maintained high AHEI scores or improved their scores during the 10-y measurement period had 65% (OR: 0.35%; 95% CI: 0.19%, 0.64%) and 68% (OR: 0.32%; 95% CI: 0.13%, 0.78%) lower odds of subsequent recurrent depressive symptoms than did women who maintained low AHEI scores. Among AHEI components, vegetable, fruit, trans fat, and the ratio of polyunsaturated fat to saturated fat components were associated with recurrent depressive symptoms in women. | Poor diet is a risk factor for future depression in women. |
| Chocano-Bedoya et al (2013)^S17^ | After adjustment for age, body mass index, and other potential confounders, no significant association was shown between the diet patterns and depression risk under the strict definition. Under the broad definition, women with the highest scores for the Western pattern had 15% higher risk of depression (95% CI 1.04–1.27; p trend = 0.01) than did women with the lowest scores, but after additional adjustment for psychological scores at baseline, results were no longer significant (RR 1.09; 95% CI 0.99–1.21; p trend = 0.08). | Results do not support a clear association between dietary patterns from factor analysis and depression risk. |
| Ford et al (2013)^S18^ | Intake of vegetables (β=0.124, 95% CI 0.101, 0.147), fruit (β=0.066, 95% CI 0.046, 0.085), olive oil (β=0.070, 95% CI 0.029, 0.111), nuts (β=0.054, 95% CI 0.026, 0.082), and legumes (β=0.055, 95% CI 0.032, 0.077) were associated with positive affect while sweets/ desserts (β=−0.066, 95% CI −0.086, −0.046), soda (β=−0.025, 95% CI −0.037, −0.013) and fast food frequency (β=−0.046, 95% CI −0.062, −0.030) were inversely associated with positive affect. Intake of sweets/desserts (β=0.058, 95% CI 0.037, 0.078) and fast food frequency (β=0.052, 95% CI 0.036, 0.068) were associated with negative affect while intake of vegetables (β=−0.076, 95% CI −0.099, −0.052), fruit (β=−0.033, 95% CI −0.053, −0.014) and nuts (β= −0.088, 95% CI −0.116, −0.060) were inversely associated with negative affect. | Foods typical of Mediterranean diets were associated with positive affect as well as lower negative affect while Western foods were associated with low positive affect in general and negative affect in women. |
| Nanri et al (2013)^S19^ | Among both men and women, a ‘prudent’ dietary pattern characterised by a high intake of vegetables, fruits, potatoes, soy products, mushrooms, seaweed and fish was associated with a decreased risk of suicide. The multivariable-adjusted hazard ratio of suicide for the highest v. lowest quartiles of the dietary pattern score was 0.46 (95% CI 0.28–0.75) (P for trend, 0.005). Other dietary patterns (Westernised and traditional Japanese) were not associated with suicide risk. | A prudent dietary pattern, may be associated with a decreased risk of suicide. |
| Rashidkhani et al (2013)^S20^ | After adjusting for confounders, individuals who gained higher scores in healthy dietary pattern, had 84% lower odds of major depression; while the odds of major depression in participants who gained higher scores in unhealthy dietary pattern showed no significant association. | Healthy dietary pattern is significantly associated with lower odds of major depression in adult women. |
| Rienks et al (2013)^S21^ | A higher consumption of the Mediterranean-style diet had a cross-sectional association with lower prevalence of depressive symptoms in 2001, adjusted odds ratio 0.82 (95% confidence interval 0.77–0.88); and longitudinally with lower incidence of depressive symptoms in 2004, adjusted odds ratio 0.83 (0.75–0.91). A dose–response relationship was found cross-sectionally when women were grouped according to quintiles of Mediterranean-style diet (p value for trend < 0.001). | Consumption of a “Mediterranean-style” dietary pattern by mid-aged women may have a protective influence against the onset of depressive symptoms. |
| Le Port et al (2012)^S22^ | The highest quartile of low-fat and high snack diets in women were associated with higher likelihood of depressive symptoms at the start of the follow-up compared to the lowest quartile. Conversely, the highest quartile of traditional diet was associated in women with a lower likelihood of depressive symptoms compared to the lowest quartile, with OR = 0.63 [95% CI, 0.50–0.80], as the healthy pattern (characterized by vegetables consumption) with OR = 0.75 [95% CI, 0.61–0.93]. However, there was probably a reverse causality effect for the healthy pattern. | Longitudinal study shows that several dietary patterns are associated with depressive symptoms and these associations track over time. |
| Chatzi et al (2011)^S23^ | High adherence to a “health conscious” diet was associated with lower EPDS scores (highest v. lowest tertile: β-coefficient = -1.75, p = 0.02). Women in the second (relative risk (RR) = 0.52, 95% CI 0.30–0.92) or third tertile (RR = 0.51, 95% CI 0.25–1.05) of the “health conscious” dietary pattern were about 50% less likely to have high levels of PPD symptoms (EPDS≥13) compared with those in the lowest tertile. | Healthy diet during pregnancy is associated with reduced risk for postpartum depression. |
| Jacka et al (2011)^S24^ | After adjustments for variables including age, education, income, physical activity, smoking, and alcohol consumption, an a priori healthy diet quality score was inversely related to depression (odds ratio [OR] = 0.71, 95% confidence interval [CI] = 0.59–0.84) and anxiety (OR 0.77, 95% CI = 0.68–0.87) in women. Women scoring higher on a healthy dietary pattern were less likely to be depressed (OR 0.68, 95% CI 0.57–0.82) or anxious (OR 0.87, 95% CI 0.77–0.98). A traditional Norwegian dietary pattern was also associated with reduced depression in women (OR 0.77, 95% CI 0.64–0.92). A western-type diet was associated with increased anxiety in women (OR 1.29, 95% CI 1.17–1.43) before final adjustment for energy intake. | Individuals with better quality diets were less likely to be depressed, whereas a higher intake of processed and unhealthy foods was associated with increased anxiety. |
| Okubo et al (2011)^S25^ | After adjustment for potential confounders, neither the „Healthy” nor the “Japanese” pattern was related to the risk of postpartum depression. | No substantiate clear association between dietary patterns and the risk of postpartum depression was observed. |
| Jacka et al (2010)^S26^ | After adjustments for age, socio­economic status, education, and health behaviors, a “traditional” dietary pattern was associated with lower odds for major depression or dysthymia and for anxiety disorders. A “western” diet was associated with a higher GHQ-12 score. There was also an inverse associa­tion between diet quality score and GHQ- 12 score that was not confounded by age, socioeconomic status, education, or other health behaviors. | The results demonstrate an association between habitual diet quality and the high-prevalence mental disorders, although reverse causality and confounding cannot be ruled out as ex­planations. |
| Beydoun et al (2009)^S27^ | The CES-D score was associated with lower HEI scores in all sex-ethnicity groups of women, except with higher waist-to-hip ratios (WHRs) among African American women. | The study suggests major sex and ethnic differences in the pathways linking SES, depressive symptoms, and lifestyle factors to central adiposity measures. In some groups and for particular adiposity measures (e.g., African American women and WHR), unhealthy eating may not be the factor responsible for the association between depression and central adiposity, depressive symptoms and unhealthy eating may both contribute to SES disparities in central adiposity for other groups and adiposity measures (e.g., white women, WC, and WHR). |
| Muñoz et al (2009)^S28^ | Age-adjusted linear regression analysis revealed a significant (p<0.01) direct association of the MDS with self-reported mental and physical health in both sexes. An increase of 5 units of the MDS was directly associated with changes of 1.15 units in women in the mental component score after controlling for potential confounders. The age-adjusted direct association of the MDS with self-reported scoring of physical health after adjusting for several confounders was attenuated in women. | Adherence to the Mediterranean diet was associated with higher scoring for self-perceived health |
| Samieri et al (2008)^S29^ | A “healthy” cluster in women (n = 267; 24.8%) had significantly lower mean number of errors to Mini Mental State score after adjustment for socio-demographic variables (β = -0.13; 95% CI -0.22 to -0.04 in women). The same cluster was associated with borderline significance with lower depressive symptoms in women (β = -0.16; 95% CI -0.33–0.007). Women in the “biscuits and snacking” cluster had greater risk of poor perceived health (OR=1.69; 95% CI 1.15–2.48) compared to “healthy” eaters. Additional adjustment for body mass index and medication use strengthened these associations. | Sex-specific dietary patterns derived by hybrid clustering method are associated with fewer depressive symptoms and better perceived health in older people. |
| Yannakoulia et al (2008)^S30^ | More anxious, compared to less anxious women exhibited different dietary patterns. In particular, the ‘‘light’’ dietary patterns that were emerged in the less anxious women did not appear as distinct patterns among women in the upper anxiety tertile. In women, a ‘‘Western-type’’ diet explained two times greater variance of food intake of those in the upper-anxiety tertile, compared to their counterparts in the low tertile. A vegetarian pattern was found only among the less anxious women, who also exhibited the lowest consumption of red meat and sweets. | More anxious, compared to less anxious, men and women exhibited different dietary patterns. |

**References**

S1**.** Boldt P, Knechtle B, Nikolaidis P, et al. Quality of life of female and male vegetarian and vegan endurance runners compared to omnivores - results from the NURMI study (step 2). *J Int Soc Sports Nutr.* 2018;15(1):33. doi:10.1186/s12970-018-0237-8.

S2. Forestell CA, Nezlek JB. Vegetarianism, depression, and the five factor model of personality. *Ecol Food Nutr.* 2018;57(3):246‐259. doi:10.1080/03670244.2018.1455675.

S3. Gomes AP, Oliveira Bierhals I, Gonçalves Soares AL, et al. Interrelatioship between Diet Quality and Depressive Symptoms in Elderly. *J Nutr Health Aging.* 2018;22(3):387‐392. doi:10.1007/s12603-017-0963-7.

S4. Li XD, Cao HJ, Xie SY, et al. Adhering to a vegetarian diet may create a greater risk of depressive symptoms in the elderly male Chinese population. *J Affect Disord.* 2019;243:182‐187. doi:10.1016/j.jad.2018.09.033.

S5. Miyake Y, Tanaka K, Okubo H, et al. Dietary patterns and depressive symptoms during pregnancy in Japan: Baseline data from the Kyushu Okinawa Maternal and Child Health Study. *J Affect Disord.* 2018;1(225):552-558. doi: 10.1016/j.jad.2017.08.073.

S6. Teo C, Chia AR, Colega MT, et al. Prospective Associations of Maternal Dietary Patterns and Postpartum Mental Health in a Multi-Ethnic Asian Cohort: The Growing up in Singapore towards Healthy Outcomes (GUSTO) Study. *Nutrients*. 2018;2;10(3):299. doi:10.3390/nu10030299.

S7. Adjibade M, Assmann KE, Andreeva VA, et al. Prospective association between adherence to the Mediterranean diet and risk of depressive symptoms in the French SU.VI.MAX cohort. *Eur J Nutr.* 2018;57(3):1225‐1235. doi:10.1007/s00394-017-1405-3.

S8. Baskin R, Hill B, Jacka FN, et al. Antenatal dietary patterns and depressive symptoms during pregnancy and early post-partum. *Matern Child Nutr.* 2017;13(1):e12218. doi:10.1111/mcn.12218. doi:10.1111/mcn.12218.

S9. Paskulin JTA, Drehmer M, Olinto MT, et al. Association between dietary patterns and mental disorders in pregnant women in Southern Brazil. *Braz J Psychiatry.* 2017;39(3):208‐215. doi:10.1590/1516-4446-2016-2016.

S10. Sakai H, Murakami K, Kobayashi S, et al. Food-based diet quality score in relation to depressive symptoms in young and middle-aged Japanese women. *Br J Nutr.* 2017;117(12):1674-1681. doi:10.1017/S0007114517001581.

S11. Huddy R.L, Torres S.J, Milte C.M, et al. Higher Adherence to the Australian Dietary Guidelines Is Associated with Better Mental Health Status among Australian Adult First-Time Mothers. *J Acad Nutr Diet.* 2016;116(9):1406‐1412. doi:10.1016/j.jand.2016.

S12. Kim WK, Shin D, Song WO. Are Dietary Patterns Associated with Depression in U.S. Adults? *J Med Food.* 2016;19(11):1074‐1084. doi:10.1089/jmf.2016.0043.

S13. Liu ZM, Ho SC, Xie YJ et al. Associations between dietary patterns and psychological factors: a cross-sectional study among Chinese postmenopausal women. *Menopause.* 2016;23(12):1294‐1302. doi:10.1097/GME.0000000000000701.

S14. Hosseinzadeh M, Vafa M, Esmaillzadeh A, et al. Empirically derived dietary patterns in relation to psychological disorders. *Public Health Nutr.* 2016;19(2):204‐217. doi:10.1017/S136898001500172X.

S15. Vilela AA, Pinto Tde J, Rebelo F, et al. Association of Prepregnancy Dietary Patterns and Anxiety Symptoms from Midpregnancy to Early Postpartum in a Prospective Cohort of Brazilian Women. *J Acad Nutr Diet.* 2015;115(10):1626‐1635. doi:10.1016/j.jand.2015.01.007.

S16. Akbaraly TN, Sabia S, Shipley MJ, et al. Adherence to healthy dietary guidelines and future depressive symptoms: evidence for sex differentials in the Whitehall II study. *Am J Clin Nutr.* 2013;97(2):419-427. doi: 10.3945/ajcn.112.041582.

S17. Chocano-Bedoya PO, O'Reilly EJ, Lucas M, et al. Prospective study on long-term dietary patterns and incident depression in middle-aged and older women. *Am J Clin Nutr.* 2013;98(3):813‐820. doi:10.3945/ajcn.112.052761.

S18. Ford PA, Jaceldo-Siegl K, Lee JW, et al. Intake of Mediterranean foods associated with positive affect and low negative affect. *J Psychosom Res.* 2013;74(2):142-148. doi: 10.1016/j.jpsychores.2012.11.002.

S19. Nanri A, Mizoue T, Poudel-Tandukar K, Noda M, et al. Japan Public Health Center-based Prospective Study Group. Dietary patterns and suicide in Japanese adults: the Japan Public Health Center-based Prospective Study. *Br J Psychiatry.* 2013;203(6):422‐427. doi:10.1192/bjp.bp.112.114793.

S20. Rashidkhani B, Pourghassem Gargari B, Ranjbar F, et al. Dietary patterns and anthropometric indices among Iranian women with major depressive disorder. *Psychiatry Res.* 2013;210(1):115‐120. doi:10.1016/j.psychres.2013.05.022.

S21. Rienks J, Dobson AJ, Mishra GD. Mediterranean dietary pattern and prevalence and incidence of depressive symptoms in mid-aged women: results from a large community-based prospective study. *Eur J Clin Nutr.* 2013;67(1):75‐82. doi:10.1038/ejcn.2012.193.

S22. Le Port A, Gueguen A, Kesse-Guyot E, et al. Association between dietary patterns and depressive symptoms over time: a 10-year follow-up study of the GAZEL cohort. *PLoS One.* 2012;7(12):e51593. doi:10.1371/journal.pone.0051593.

S23. Chatzi L, Melaki V, Sarri K, et al. Dietary patterns during pregnancy and the risk of postpartum depression: the mother-child 'Rhea' cohort in Crete, Greece. P*ublic Health Nutr.* 2011;14(9):1663‐1670. doi:10.1017/S1368980010003629.

S24. Jacka FN, Mykletun A, Berk M, et al. The association between habitual diet quality and the common mental disorders in community-dwelling adults: the Hordaland Health study. *Psychosom Med.* 2011;73(6):483‐490. doi:10.1097/PSY.0b013e318222831a.

S25. Okubo H, Miyake Y, Sasaki S, et al. Osaka Maternal and Child Health Study Group. Dietary patterns during pregnancy and the risk of postpartum depression in Japan: the Osaka Maternal and Child Health Study. *Br J Nutr.* 2011;105(8):1251‐1257. doi:10.1017/S0007114510004782.

S26. Jacka FN, Pasco JA, Mykletun A, et al. Association of Western and traditional diets with depression and anxiety in women. *Am J Psychiatry.* 2010;167(3):305‐311. doi:10.1176/appi.ajp.2009.09060881.

S27. Beydoun MA, Kuczmarski MT, Mason MA, et al. Role of depressive symptoms in explaining socioeconomic status disparities in dietary quality and central adiposity among US adults: a structural equation modeling approach. *Am J Clin Nutr.* 2009;90(4):1084‐1095. doi:10.3945/ajcn.2009.27782.

S28. Muñoz MA, Fíto M, Marrugat J, et al. Adherence to the Mediterranean diet is associated with better mental and physical health. *Br J Nutr.* 2009;101(12):1821‐1827. doi:10.1017/S0007114508143598.

S29. Samieri C, Jutand MA, Féart C, et al. Dietary patterns derived by hybrid clustering method in older people: association with cognition, mood, and self-rated health. *J Am Diet Assoc.* 2008;108(9):1461‐1471. doi:10.1016/j.jada.2008.06.437.

S30. Yannakoulia M, Panagiotakos DB, Pitsavos C, et al. Eating habits in relations to anxiety symptoms among apparently healthy adults. A pattern analysis from the ATTICA Study. *Appetite.* 2008;1(3):519‐525. doi:10.1016/j.appet.2008.04.002.

**Table 5.** The NOS scores for the categories of selection, comparability, and exposure/outcome, the assessment of the quality of included studies.

| **Ref**. | **Scores for categories** | | | **Quality** | |
| --- | --- | --- | --- | --- | --- |
|  | **Selection** | **Comparability** | **Exposure/outcome** | **Total score^a^** | **Risk of bias** |
| Boldt et al (2018)^S1^ | 3 | 2 | 3 | 8 | Low |
| Forestell and Nezlek (2018)^S2^ | 4 | 2 | 3 | 9 | Low |
| Gomes et al (2018)^S3^ | 3 | 2 | 2 | 7 | Low |
| Li et al (2018)^S4^ | 2 | 2 | 1 | 5 | High |
| Miyake et al (2018)^S5^ | 1 | 2 | 0 | 3 | Very high |
| Teo et al (2018)^S6^ | 4 | 2 | 3 | 9 | Low |
| Adjibade et al (2017)^S7^ | 4 | 2 | 2 | 8 | Low |
| Baskin et al (2017)^S8^ | 2 | 2 | 1 | 5 | High |
| Paskulin et al (2017)^S9^ | 2 | 1 | 2 | 5 | High |
| Sakai et al (2017)^S10^ | 3 | 2 | 2 | 7 | Low |
| Huddy et al (2016)^S11^ | 3 | 2 | 2 | 7 | Low |
| Kim et al (2016)^S12^ | 3 | 2 | 2 | 7 | Low |
| Liu et al (2016)^213^ | 2 | 2 | 2 | 6 | High |
| Hosseinzadeh et al (2015)^S14^ | 3 | 2 | 2 | 7 | Low |
| Vilela et al (2015)^S15^ | 2 | 2 | 1 | 5 | High |
| Akbaraly et al (2013)^S16^ | 3 | 2 | 3 | 8 | Low |
| Chocano-Bedoya et al (2013)^S17^ | 2 | 2 | 2 | 6 | High |
| Ford et al (2013)^S18^ | 4 | 2 | 1 | 7 | Low |
| Nanri et al (2013)^S19^ | 3 | 2 | 1 | 6 | High |
| Rashidkhani et al (2013)^S20^ | 3 | 2 | 3 | 8 | Low |
| Rienks et al (2013)^S21^ | 3 | 2 | 2 | 7 | Low |
| Le Port et al (2012)^S22^ | 3 | 2 | 2 | 7 | Low |
| Chatzi et al (2011)^S23^ | 3 | 2 | 2 | 7 | Low |
| Jacka et al (2011)^S24^ | 3 | 2 | 1 | 6 | High |
| Okubo et al (2011)^S25^ | 4 | 2 | 3 | 9 | Low |
| Jacka et al (2010)^S26^ | 3 | 2 | 2 | 7 | Low |
| Beydoun et al (2009)^S27^ | 4 | 2 | 2 | 8 | Low |
| Muñoz et al (2009)^S28^ | 2 | 2 | 3 | 7 | Low |
| Samieri et al (2008)^S29^ | 3 | 2 | 1 | 6 | High |
| Yannakoulia et al (2008)^S30^ | 2 | 2 | 2 | 6 | High |

^a^ total score for the Newcastle-Ottawa Scale (NOS) is attributed to a following categories: very high risk of bias (0-3 NOS points), high risk of bias (4-6 NOS points), and low risk of bias (7-9 NOS points) (Wells et al.^S31^).

**References**

S1**.** Boldt P, Knechtle B, Nikolaidis P, et al. Quality of life of female and male vegetarian and vegan endurance runners compared to omnivores - results from the NURMI study (step 2). *J Int Soc Sports Nutr.* 2018;15(1):33. doi:10.1186/s12970-018-0237-8.

S2. Forestell CA, Nezlek JB. Vegetarianism, depression, and the five factor model of personality. *Ecol Food Nutr.* 2018;57(3):246‐259. doi:10.1080/03670244.2018.1455675.

S3. Gomes AP, Oliveira Bierhals I, Gonçalves Soares AL, et al. Interrelatioship between Diet Quality and Depressive Symptoms in Elderly. *J Nutr Health Aging.* 2018;22(3):387‐392. doi:10.1007/s12603-017-0963-7.

S4. Li XD, Cao HJ, Xie SY, et al. Adhering to a vegetarian diet may create a greater risk of depressive symptoms in the elderly male Chinese population. *J Affect Disord.* 2019;243:182‐187. doi:10.1016/j.jad.2018.09.033.

S5. Miyake Y, Tanaka K, Okubo H, et al. Dietary patterns and depressive symptoms during pregnancy in Japan: Baseline data from the Kyushu Okinawa Maternal and Child Health Study. *J Affect Disord.* 2018;1(225):552-558. doi: 10.1016/j.jad.2017.08.073.

S6. Teo C, Chia AR, Colega MT, et al. Prospective Associations of Maternal Dietary Patterns and Postpartum Mental Health in a Multi-Ethnic Asian Cohort: The Growing up in Singapore towards Healthy Outcomes (GUSTO) Study. *Nutrients*. 2018;2;10(3):299. doi:10.3390/nu10030299.

S7. Adjibade M, Assmann KE, Andreeva VA, et al. Prospective association between adherence to the Mediterranean diet and risk of depressive symptoms in the French SU.VI.MAX cohort. *Eur J Nutr.* 2018;57(3):1225‐1235. doi:10.1007/s00394-017-1405-3.

S8. Baskin R, Hill B, Jacka FN, et al. Antenatal dietary patterns and depressive symptoms during pregnancy and early post-partum. *Matern Child Nutr.* 2017;13(1):e12218. doi:10.1111/mcn.12218. doi:10.1111/mcn.12218.

S9. Paskulin JTA, Drehmer M, Olinto MT, et al. Association between dietary patterns and mental disorders in pregnant women in Southern Brazil. *Braz J Psychiatry.* 2017;39(3):208‐215. doi:10.1590/1516-4446-2016-2016.

S10. Sakai H, Murakami K, Kobayashi S, et al. Food-based diet quality score in relation to depressive symptoms in young and middle-aged Japanese women. *Br J Nutr.* 2017;117(12):1674-1681. doi:10.1017/S0007114517001581.

S11. Huddy R.L, Torres S.J, Milte C.M, et al. Higher Adherence to the Australian Dietary Guidelines Is Associated with Better Mental Health Status among Australian Adult First-Time Mothers. *J Acad Nutr Diet.* 2016;116(9):1406‐1412. doi:10.1016/j.jand.2016.

S12. Kim WK, Shin D, Song WO. Are Dietary Patterns Associated with Depression in U.S. Adults? *J Med Food.* 2016;19(11):1074‐1084. doi:10.1089/jmf.2016.0043.

S13. Liu ZM, Ho SC, Xie YJ et al. Associations between dietary patterns and psychological factors: a cross-sectional study among Chinese postmenopausal women. *Menopause.* 2016;23(12):1294‐1302. doi:10.1097/GME.0000000000000701.

S14. Hosseinzadeh M, Vafa M, Esmaillzadeh A, et al. Empirically derived dietary patterns in relation to psychological disorders. *Public Health Nutr.* 2016;19(2):204‐217. doi:10.1017/S136898001500172X.

S15. Vilela AA, Pinto Tde J, Rebelo F, et al. Association of Prepregnancy Dietary Patterns and Anxiety Symptoms from Midpregnancy to Early Postpartum in a Prospective Cohort of Brazilian Women. *J Acad Nutr Diet.* 2015;115(10):1626‐1635. doi:10.1016/j.jand.2015.01.007.

S16. Akbaraly TN, Sabia S, Shipley MJ, et al. Adherence to healthy dietary guidelines and future depressive symptoms: evidence for sex differentials in the Whitehall II study. *Am J Clin Nutr.* 2013;97(2):419-427. doi: 10.3945/ajcn.112.041582.

S17. Chocano-Bedoya PO, O'Reilly EJ, Lucas M, et al. Prospective study on long-term dietary patterns and incident depression in middle-aged and older women. *Am J Clin Nutr.* 2013;98(3):813‐820. doi:10.3945/ajcn.112.052761.

S18. Ford PA, Jaceldo-Siegl K, Lee JW, et al. Intake of Mediterranean foods associated with positive affect and low negative affect. *J Psychosom Res.* 2013;74(2):142-148. doi: 10.1016/j.jpsychores.2012.11.002.

S19. Nanri A, Mizoue T, Poudel-Tandukar K, Noda M, et al. Japan Public Health Center-based Prospective Study Group. Dietary patterns and suicide in Japanese adults: the Japan Public Health Center-based Prospective Study. *Br J Psychiatry.* 2013;203(6):422‐427. doi:10.1192/bjp.bp.112.114793.

S20. Rashidkhani B, Pourghassem Gargari B, Ranjbar F, et al. Dietary patterns and anthropometric indices among Iranian women with major depressive disorder. *Psychiatry Res.* 2013;210(1):115‐120. doi:10.1016/j.psychres.2013.05.022.

S21. Rienks J, Dobson AJ, Mishra GD. Mediterranean dietary pattern and prevalence and incidence of depressive symptoms in mid-aged women: results from a large community-based prospective study. *Eur J Clin Nutr.* 2013;67(1):75‐82. doi:10.1038/ejcn.2012.193.

S22. Le Port A, Gueguen A, Kesse-Guyot E, et al. Association between dietary patterns and depressive symptoms over time: a 10-year follow-up study of the GAZEL cohort. *PLoS One.* 2012;7(12):e51593. doi:10.1371/journal.pone.0051593.

S23. Chatzi L, Melaki V, Sarri K, et al. Dietary patterns during pregnancy and the risk of postpartum depression: the mother-child 'Rhea' cohort in Crete, Greece. P*ublic Health Nutr.* 2011;14(9):1663‐1670. doi:10.1017/S1368980010003629.

S24. Jacka FN, Mykletun A, Berk M, et al. The association between habitual diet quality and the common mental disorders in community-dwelling adults: the Hordaland Health study. *Psychosom Med.* 2011;73(6):483‐490. doi:10.1097/PSY.0b013e318222831a.

S25. Okubo H, Miyake Y, Sasaki S, et al. Osaka Maternal and Child Health Study Group. Dietary patterns during pregnancy and the risk of postpartum depression in Japan: the Osaka Maternal and Child Health Study. *Br J Nutr.* 2011;105(8):1251‐1257. doi:10.1017/S0007114510004782.

S26. Jacka FN, Pasco JA, Mykletun A, et al. Association of Western and traditional diets with depression and anxiety in women. *Am J Psychiatry.* 2010;167(3):305‐311. doi:10.1176/appi.ajp.2009.09060881.

S27. Beydoun MA, Kuczmarski MT, Mason MA, et al. Role of depressive symptoms in explaining socioeconomic status disparities in dietary quality and central adiposity among US adults: a structural equation modeling approach. *Am J Clin Nutr.* 2009;90(4):1084‐1095. doi:10.3945/ajcn.2009.27782.

S28. Muñoz MA, Fíto M, Marrugat J, et al. Adherence to the Mediterranean diet is associated with better mental and physical health. *Br J Nutr.* 2009;101(12):1821‐1827. doi:10.1017/S0007114508143598.

S29. Samieri C, Jutand MA, Féart C, et al. Dietary patterns derived by hybrid clustering method in older people: association with cognition, mood, and self-rated health. *J Am Diet Assoc.* 2008;108(9):1461‐1471. doi:10.1016/j.jada.2008.06.437.

S30. Yannakoulia M, Panagiotakos DB, Pitsavos C, et al. Eating habits in relations to anxiety symptoms among apparently healthy adults. A pattern analysis from the ATTICA Study. *Appetite.* 2008;1(3):519‐525. doi:10.1016/j.appet.2008.04.002.

S31. Wells GA, Shea B, O’Connell D, et al. The Newcastle-Ottawa Scale (NOS) for Assessing the Quality of Nonrandomised Studies in Meta-Analyses. Accessed July 22, 2020. http://www.ohri.ca/programs/clinical_epidemiology/oxford.asp.
